# Extensive Regulation of Diurnal Transcription and Metabolism by Glucocorticoids

**DOI:** 10.1371/journal.pgen.1006512

**Published:** 2016-12-12

**Authors:** Benjamin D. Weger, Meltem Weger, Benjamin Görling, Andrea Schink, Cédric Gobet, Céline Keime, Gernot Poschet, Bernard Jost, Nils Krone, Rüdiger Hell, Frédéric Gachon, Burkhard Luy, Thomas Dickmeis

**Affiliations:** 1 Institute of Toxicology and Genetics, Karlsruhe Institute of Technology, Hermann-von-Helmholtz-Platz 1, Eggenstein-Leopoldshafen, Germany; 2 Nestlé Institute of Health Sciences SA, EPFL Innovation Park, Bâtiment H, Lausanne, Switzerland; 3 Centre for Endocrinology, Diabetes and Metabolism, University of Birmingham, Birmingham, United Kingdom; 4 Institute for Organic Chemistry, Karlsruhe Institute of Technology, Hermann-von-Helmholtz-Platz 1, Eggenstein-Leopoldshafen, Germany; 5 Institute for Biological Interfaces 4 –Magnetic Resonance, Karlsruhe Institute of Technology, Hermann-von-Helmholtz-Platz 1, Eggenstein-Leopoldshafen, Germany; 6 Faculty of Life Sciences, Ecole Polytechnique Fédérale de Lausanne, Lausanne, Switzerland; 7 Plateforme Biopuces et séquençage, IGBMC, 1 rue Laurent Fries, Parc d'Innovation, Illkirch, France; 8 Centre for Organismal Studies (COS), University of Heidelberg, Heidelberg, Germany; 9 Academic Unit of Child Health, Department of Oncology and Metabolism, University of Sheffield, Sheffield, United Kingdom; University of Lübeck, GERMANY

## Abstract

Altered daily patterns of hormone action are suspected to contribute to metabolic disease. It is poorly understood how the adrenal glucocorticoid hormones contribute to the coordination of daily global patterns of transcription and metabolism. Here, we examined diurnal metabolite and transcriptome patterns in a zebrafish glucocorticoid deficiency model by RNA-Seq, NMR spectroscopy and liquid chromatography-based methods. We observed dysregulation of metabolic pathways including glutaminolysis, the citrate and urea cycles and glyoxylate detoxification. Constant, non-rhythmic glucocorticoid treatment rescued many of these changes, with some notable exceptions among the amino acid related pathways. Surprisingly, the non-rhythmic glucocorticoid treatment rescued almost half of the entire dysregulated diurnal transcriptome patterns. A combination of E-box and glucocorticoid response elements is enriched in the rescued genes. This simple enhancer element combination is sufficient to drive rhythmic circadian reporter gene expression under non-rhythmic glucocorticoid exposure, revealing a permissive function for the hormones in glucocorticoid-dependent circadian transcription. Our work highlights metabolic pathways potentially contributing to morbidity in patients with glucocorticoid deficiency, even under glucocorticoid replacement therapy. Moreover, we provide mechanistic insight into the interaction between the circadian clock and glucocorticoids in the transcriptional regulation of metabolism.

## Author Summary

Glucocorticoids, steroid hormones of the adrenal gland, are important regulators of metabolism and the stress response. They are also widely used as anti-inflammatory drugs. Production and release of glucocorticoids show a diurnal pattern regulated by the circadian clock. Importantly, altered daily patterns of hormone action are thought to contribute to metabolic diseases. Here, we examined diurnal patterns of gene expression and metabolism in a zebrafish model of glucocorticoid deficiency. We observed that a surprisingly large number of genes show glucocorticoid-dependent diurnal patterns of transcription. This behaviour is particularly pronounced in metabolic genes, and metabolites of various central metabolic pathways are dysregulated. Interestingly, non-rhythmic glucocorticoid treatment restored many of these metabolite changes. It also restored expression of almost half of the dysregulated genes. Using *in vivo* bioluminescence assays, we provide evidence that this rescue is mediated via combined glucocorticoid–circadian clock activity on a simple regulatory DNA module enriched in the genomic vicinity of these genes. Our work provides mechanistic insight into how the circadian clock and glucocorticoids cooperate in the regulation of daily patterns of gene expression and metabolism. Furthermore, it highlights metabolic pathways that may contribute to disease mechanisms in patients with a disturbed glucocorticoid hormone system.

## Introduction

The circadian clock is an endogenous oscillator that regulates daily changes of behavior, physiology and metabolism [1]. The molecular basis of the circadian clock is a transcriptional-translational feedback loop, a central part of which are E-box enhancer elements [2]. To generate physiological rhythms, “peripheral” clocks in almost all tissues interact with signals produced by a “central” pacemaker, the hypothalamic suprachiasmatic nucleus. A key target of circadian clock control is metabolism, with circadian rhythms present in many metabolites and enzyme activities [3]. In addition, hormones with metabolic functions are regulated by the circadian clock. This includes glucocorticoids (GCs), steroid hormones mainly produced by the adrenal gland [4]. GC production shows higher basal levels in the morning in humans and at night in rodents. GCs were also shown to interact with clock factors in the transcriptional regulation of metabolic gene expression [5]. However, the global role of the interaction between the circadian clock and GCs in the regulation of physiology and metabolism and its underlying mechanisms are only incompletely understood.

Patients suffering from adrenal insufficiency (AI) have inadequate GC amounts either because of defects in the adrenal gland itself (primary AI) or due to deficient input from the pituitary gland (secondary AI) [6]. Patients with secondary AI have an increased risk to develop metabolic syndrome, and abnormal glucose tolerance is observed upon long-term therapy with current GC replacement regimes. This may be linked to an inadequate replication of the natural circadian GC rhythm [7]. Only limited information is available on the metabolic changes present in patients with AI [8,9], particularly with respect to their temporal dynamics. Animal models, preferentially with a diurnal lifestyle, could contribute to improve therapy by providing a mechanistic understanding of metabolic dysregulation in AI.

**Fig 1:**
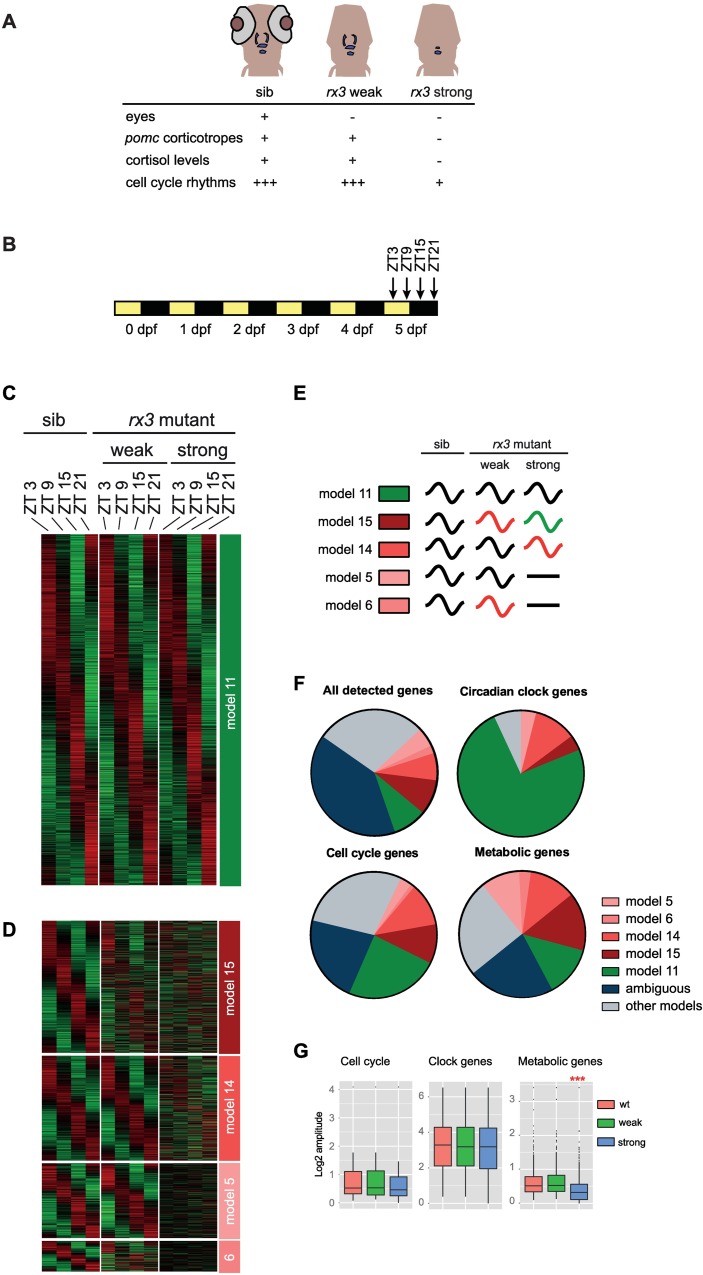
Glucocorticoids regulate a large part of the diurnal transcriptome. (A) *rx3* mutant phenotypes. Blue: *pomc* expression in larval head. sib: wild-type sibling. (B) Sampling schedules of the transcriptomics studies. Zebrafish embryos/larvae were raised under 12 h light (yellow) / 12 h dark (black) cycles. dpf: days post fertilization. ZT: *Zeitgeber* Time in hours after lights on (ZT0); lights off: ZT12. (C,D) Heatmaps of normalized mRNA expression levels of model 11 (C) and model 5-6-14-15 (D) genes. Red, high expression. Green, low expression. Expression levels normalized by the mean of each gene within each phenotype combination. (E) Overview of gene expression profiles in the different models. Black curved line: oscillation with same phase and amplitude as wild-type, red or green curved lines: oscillation with a phase or amplitude different from wild-type, black straight line: no oscillation. The green curved line indicates an oscillation with an amplitude and/or phase that is both different from wild-type (black) and from the other changed oscillation (red). (F) Model distribution among genes of different functional categories. (G) Tukey boxplots depicting amplitudes of gene expression oscillations in the indicated functional categories. Red stars, significant amplitude change (adjusted *p*-value = 2.2e-16).

The zebrafish is a well-established model system for human disease including metabolic diseases [10] and has proven useful for chronobiology and endocrinology studies [11,12]. Zebrafish embryos and larvae are well-suited for *in vivo* bioimaging and drug screenings [13]. Similar to humans, zebrafish are diurnal and use cortisol as the main GC hormone, whereas laboratory rodents are nocturnal and use corticosterone. We previously described a mutation that leads to GC deficiency in homozygous larvae. *rx3* mutants of both a weak and a strong allele show a severe eye defect [14]. The strong allele additionally presents a severe reduction of ACTH producing corticotrope pituitary cells, leading to reduced cortisol amounts which also lack a diurnal rhythm (Fig 1A)[15]. Thus, this mutant condition resembles secondary AI. Intriguingly, a clock output rhythm, the circadian fluctuation of cell proliferation, is attenuated in the mutant larvae. These rhythms can be rescued by constant treatment with the synthetic GC, dexamethasone (DEX) [15], and are thus not dependent on the diurnal glucocorticoid release pattern. It is currently not understood how a constant GC signal integrates with circadian clock function to generate such GC-dependent clock output rhythms.

Here, we examined diurnal changes in the transcriptome and metabolism of *rx3 strong* mutants with or without continuous DEX exposure. A surprisingly large part of diurnal gene expression is rescued by this constant DEX treatment, which also relieves specific metabolite changes in the mutants. Analysis of gene regulation revealed a combined simple enhancer element that is sufficient to mediate diurnal GC-dependent transcription. Besides providing mechanistic insight on GC-circadian clock crosstalk, our study reveals widespread changes in metabolism in an animal model of GC deficiency. These findings will help to better understand morbidities in patients with AI and identify metabolic pathways that could be used for monitoring of therapy efficiency.

## Results

### A large part of diurnal transcriptome changes is dependent on GCs

To examine if and how diurnal patterns of transcription are perturbed in the GC deficiency model, we measured diurnal transcriptome changes in *rx3 strong* mutant zebrafish larvae and their wild-type siblings (Fig 1A) at four time points over 24 h (Fig 1B; S1 Table, for quality control and validation experiment results see S1A–S1C Fig). To control for eye absence in the strong allele, we included the equally eyeless *rx3 weak* mutant larvae, which have normal diurnal cortisol levels. Statistical analysis based on harmonic linear regression [16] grouped genes into models according to their rhythmic or non-rhythmic expression behaviour under the three conditions (Fig 1C–1E, S2A Fig, S1 Table; see Materials and Methods for details). Genes classified into model 1 did not exhibit rhythmicity under any condition, while genes grouped within the other 14 models showed rhythmic expression in at least one condition. Genes which did not fulfill our statistical cutoff criteria to fit within these models were named “ambiguous”. Several models were of particular interest for our aim to identify genes with GC dependent diurnal patterns of transcription. Model 11 was rhythmic in all conditions with the same rhythmic parameters (Fig 1C and 1E); we will refer to this group of genes as “unaffected”. By contrast, models 5, 6, 14 and 15 showed either a lack of rhythmic expression (model 5, 6) or a change in amplitude or phase (model 14, 15) in the strong allele (Fig 1D and 1E). For these four models, global gene expression amplitudes are not significantly different between wild-type and *rx3 weak* allele mutants, while they are significantly reduced (model 14 and 15) or absent (model 5 and 6) compared with the wild-type in strong allele mutants (S2D–S2G Fig). Furthermore, global comparison of phases shows that they are more perturbed when comparing wild-type and strong allele mutants than when comparing wild-type and weak allele mutants (S2H–S2K Fig). We will refer to the genes of models 5, 6, 14 and 15 as “affected”. These four models define a set of 5970 genes that are candidates for mediating GC-dependent circadian functions. Representing 43.6% of all genes showing rhythmic expression and 23.4% of all detected genes, they constitute a surprisingly large category of the diurnal transcriptome (Fig 1F). Gene Ontology (GO) analysis showed enrichment in GO terms for metabolic processes in this set (S1E Fig). Indeed, 39.9% of the temporal profiles of metabolic genes are assigned to model 5-6-14-15 genes (Fig 1F). By contrast, circadian clock and cell cycle genes are less affected in their temporal expression in *rx3 strong* mutants. 75% of all circadian clock genes are not altered in their rhythmicity and belong to model 11 (Fig 1F, S3A Fig). There is also enrichment for model 11 within the group of cell cycle genes (23.8%, Fig 1F, S3B Fig). Fitting this observation, model 11 genes are enriched for GO terms related to the cell cycle (S1D Fig). These findings show that many cell cycle related genes have a rhythmic expression pattern which does not change in the *rx3 strong* mutants. Still, 25.4% of cell cycle genes belong to model 5-6-14-15 (Fig 1F). Interestingly, there is no statistically significant alteration in oscillation amplitude between weak and strong allele mutants across both all cell cycle genes and all circadian clock genes (Fig 1G). However, the amplitudes of oscillation are significantly reduced across the metabolic genes in *rx3 strong* mutants (Fig 1G), further indicating a higher degree of attenuated rhythm within this group. The “affected” set of genes also encompasses a larger number of enriched metabolic pathways than the “unaffected” set (compare S1F Fig with S1G Fig), again underlining the strong effect present in this group on the diurnal expression of metabolic genes.

### GC treatment partially restores diurnal gene transcription

**Fig 2:**
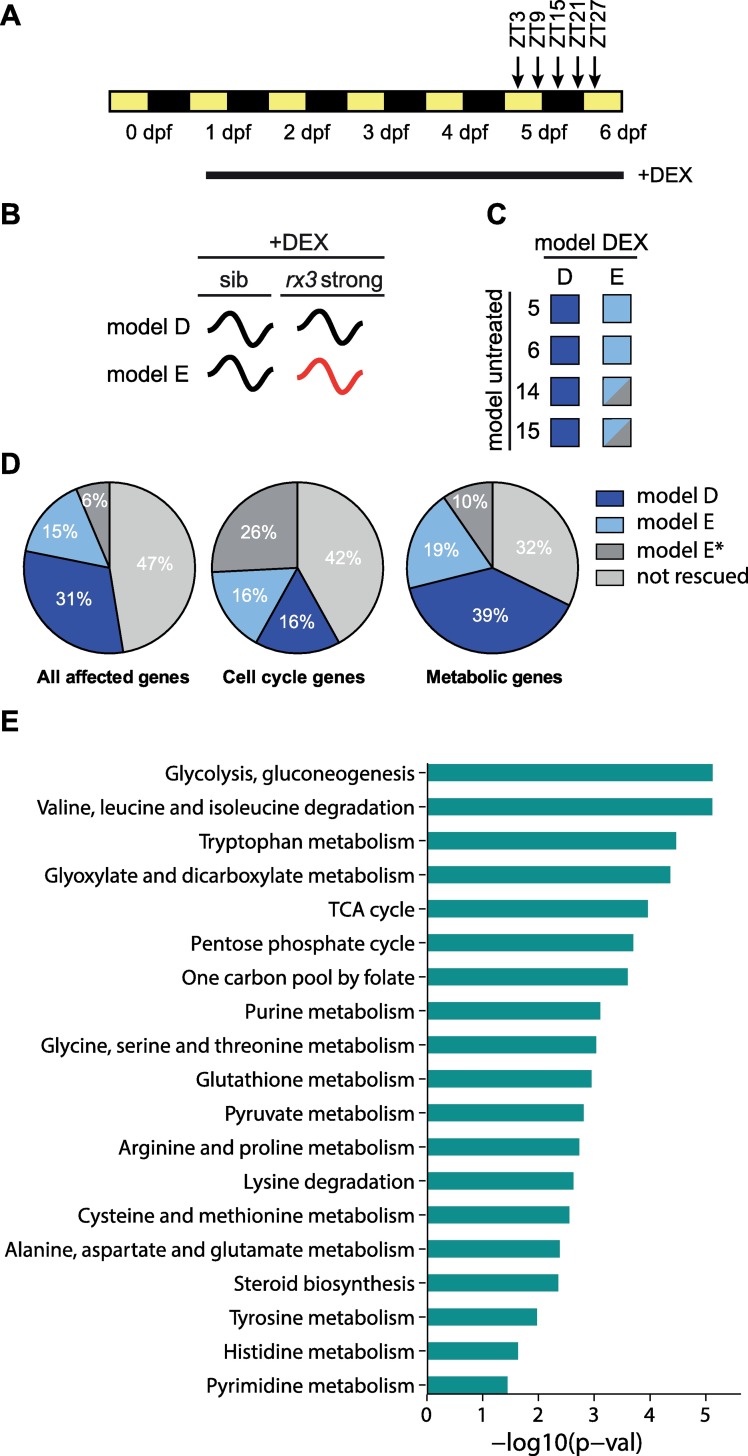
Constant DEX treatment rescues diurnal gene expression patterns especially of metabolic genes. (A) Sampling and treatment schedules of the transcriptomics and metabolomics studies. Zebrafish embryos/larvae were raised under 12 h light (yellow) / 12 h dark (black) cycles. dpf: days post fertilization. ZT: *Zeitgeber* Time in hours after lights on (ZT0); lights off: ZT12. DEX: dexamethasone. (B) Model assignments under DEX rescue. Black curved line: oscillation with same phase and amplitude as wild-type, red curved line: oscillation with a phase or amplitude different from wild-type. (C) Genes defined as rescued. Rescued genes of all “affected” models in the untreated situation (5,6,14,15) that are now classified as model D under DEX treatment (= showing identical phase and amplitude in both phenotypes) are indicated as dark blue. Genes classified as model E under DEX treatment that were either not rhythmic in the untreated mutant situation (model 5 and 6) or that were rhythmic (models 14 and 15), but showed higher phase or amplitude differences in the untreated than in the treated situation, are indicated as light blue. Genes of model 14 and 15 now classified as model E, but with no reduction of phase or amplitude differences are named model E* and indicated by dark grey. Model E* genes are not considered as rescued. (D) Pie charts depicting the behaviour of model 5-6-14-15 genes under DEX treatment. Genes not rescued by DEX treatment are indicated by the dark (model E*) and light (models other than D or E) grey colours. (E) Enriched metabolic pathways among the rescued genes.

Next, we asked whether and to what extent affected gene expression rhythms can be restored in the mutants by constant GC treatment. To determine how the diurnal transcriptome changes under chronic DEX treatment (Fig 2A), we carried out RNA-Seq analysis of treated *rx3 strong* mutants and wild-type siblings. To evaluate whether the treatment leads to a rescue of rhythmic expression in the mutants, we analyzed the two treated conditions for rhythmic parameters of gene expression as done for the untreated samples. This allowed us to evaluate if differences in expression between the genotypes were abolished by the treatment, even if other general DEX effects on transcription affected both conditions similarly. Our statistical analysis yielded five models (S2B Fig), of which two (D and E) exhibit rhythmicity in both wild-types and mutants. Therefore, these models could indicate a rescue of dysregulated patterns among the “affected” gene group (Fig 2B and 2C; S4 Fig). Model D regroups those genes in which there is no difference in rhythmic expression between mutants and wild-type under DEX treatment. Model E indicates those genes in which a difference in phase or amplitude between mutant and wild-type is still present under DEX treatment. We classified as rescued all genes which were not rhythmic in the mutants before (model 5 and 6) and in which rhythms have been restored (model D or E), or genes of models 14 and 15 in which a phase or relative amplitude difference in the untreated condition has been abolished (model D) or reduced (model E). By contrast, we did not count as rescued all those genes of model E in which phase or amplitude differences were not reduced by the treatment (named model E*).

Applying these rescue criteria, 46% of the model 5-6-14-15 genes were rescued (Fig 2D, “all affected genes”). Strikingly, 58% of the metabolic genes of model 5-6-14-15 are rescued (Fig 2D). Interestingly, even though 68% of affected cell cycle genes do not show convergence of mutant and wild-type expression patterns in the DEX treated condition (Fig 2D, S3B Fig), this treatment restores cell cycle rhythms in the *rx3 strong* mutants [15]. In summary, chronic DEX treatment is able to restore rhythmic expression patterns matching the wild-type in nearly half of all rhythmic genes affected in the mutants and in about 60% of the metabolic genes. This is a striking finding, showing that a large proportion of affected genes do not require *rhythmic* GC input for the GC-dependent regulation of their rhythmic transcription.

### Cortisol deficient *rx3 strong* mutants show diurnal metabolome changes that correlate with changes in transcription

**Fig 3:**
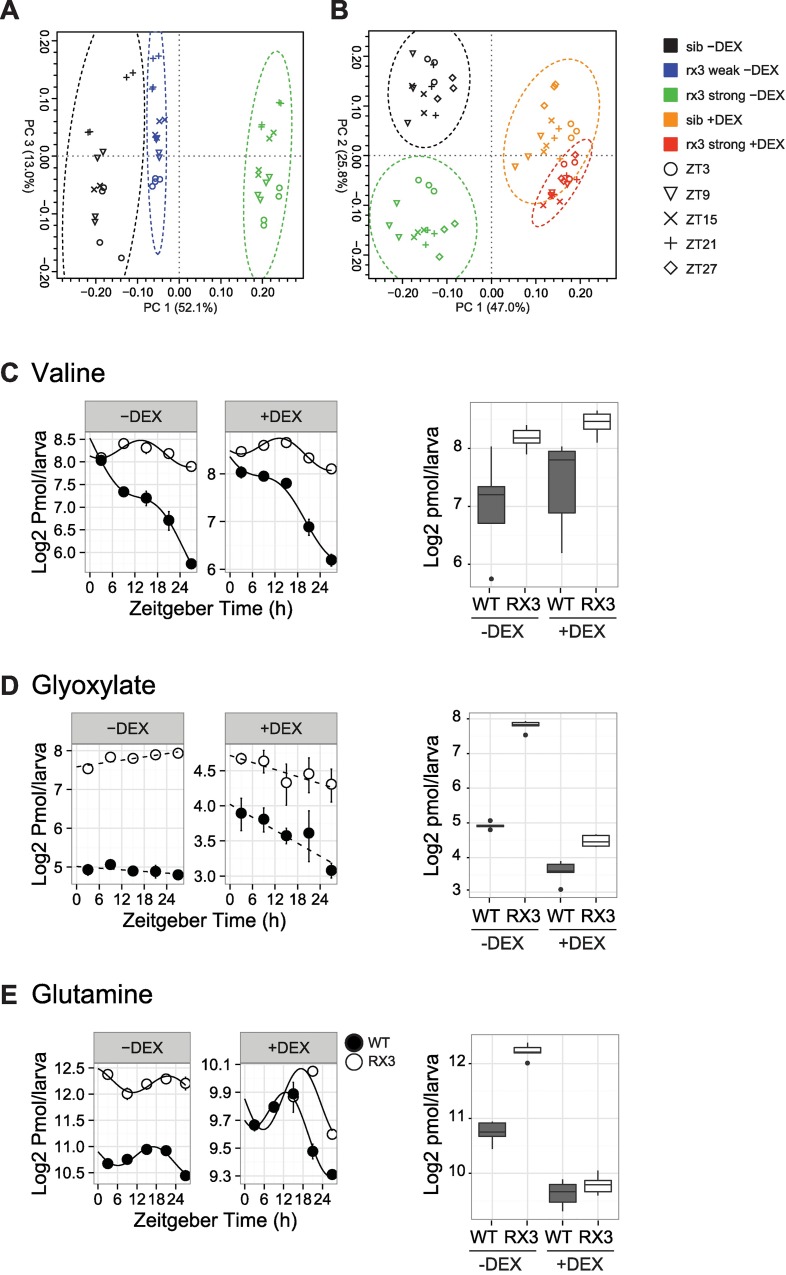
Diurnal metabolite patterns show differential dependence on glucocorticoids. (A, B) Principal component analysis (PCA) score plots of the NMR spectra of untreated samples (A) and of DEX treated together with control samples (B). (C-E) Left: Temporal profiles of metabolite levels determined by UPLC-FLR for the indicated phenotype/treatment combinations. Lines fitted by linear regression if statistical models indicate rhythmicity for wild-type (black) and *rx3 strong* mutant samples (white). Rhythmic model fits are indicated as continuous lines, non-rhythmic fits as broken lines. Error bars represent SEM. Right: Tukey boxplots depicting metabolite levels at all examined time points in wild-type siblings (WT, white) and *rx3 strong* mutants (RX3, grey) in the absence or presence of DEX.

Our RNA-Seq analysis revealed a high proportion of metabolism-related genes among the set with GC-dependent diurnal transcription. Therefore, to determine metabolite changes, we examined extracts from *rx3 strong* and *rx3 weak* mutant zebrafish larvae and their wild-type siblings at five time points over 24 h (Fig 2A) by NMR spectroscopy. We recorded 1D spectra for quantitation and additionally 2D J-resolved spectra for unambiguous identification of compounds. Principal component analysis (PCA) of the NMR spectra (Fig 3A) shows that *rx3 weak* and wild-type samples cluster together, illustrating that the metabolomes of *rx3 weak* mutants and wild-type larvae are more similar to each other than to the strong allele samples. Under DEX treatment, the wild-type and *rx3 strong* mutants cluster much closer together than the control samples (Fig 3B). Betaine, creatine, lactate and glutamine appear as major contributors to the main principal components (S5A and S5B Fig). Indeed, glutamine showed a strong accumulation in *rx3 strong* larvae at all examined time points, which was rescued by DEX treatment (S5C Fig).

Glutamine plays an important role in amino acid and central carbon metabolism, pathways which were also found to be enriched among the rescued gene set (Fig 2E). Therefore, we measured a set of amino acids and TCA cycle related metabolites by UPLC-FLR and IC-CD analysis, and evaluated rhythmicity of these data using the harmonic linear regression based model selection approach (S2C Fig, S2 Table). Rhythmicity behaviour and changes in mean levels of the metabolites indicated three groups of particular interest.

In the first group, which includes branched chain amino acids (BCAAs) and aromatic amino acids (AAAs), there is an accumulation of compounds in *rx3 strong* mutants which is not rescued by DEX treatment (Fig 3C, S5D–S5G Fig and S2 Table). Generally, compound levels in the wild-type show a stronger overall decrease over time than in the mutants. Also, rhythmicity behaviour in this group is not dramatically affected by GCs (models II [Tyr] and VI [Val, Leu, Ile, Phe]). These findings only partly correlate with gene expression pattern changes in the first degradation steps of the corresponding pathways. In the AAA pathway, half of the affected genes are rescued (S6A Fig), and in the BCAA pathway, all three key enzymes are rescued (S6B Fig). Here, metabolite accumulation seems to reflect other processes that are independent of GC regulation or not rescued by constant DEX application. Such processes may include posttranscriptional and -translational regulation of these enzymes, or BCAA accumulation due to increased degradation of BCAA containing proteins.

The second group of metabolites equally accumulates in the mutants, but here their levels are rescued by DEX treatment. Glyoxylate, lysine and the ornithine-urea cycle (OUC) compounds ornithine, citrulline and arginine belong to this group (Fig 3D, S5H–S5K Fig and S2 Table). Rhythmicity behavior varies in this group (models I [glyoxylate], III [Cit], VII [Arg] or ambiguous [Lys, Orn]). Patterns of gene expression in the corresponding pathways seem to be more closely correlated with metabolite changes than in the first group: glyoxylate metabolizing enzymes are downregulated, while those producing glyoxylate are upregulated in *rx3 strong* mutants, and changes in both pathways are rescued by DEX treatment (S6C Fig). A similar behavior is seen in the OUC pathway (S6D Fig).

The last group contains only one compound, glutamine. Here, accumulation in *rx3 strong* is rescued by DEX treatment, as in the second group. Additionally, glutamine shows a diurnal rhythm in the wild-type which is slightly flattened and shifted in the *rx3 strong* mutant (log2 amplitude 0.40 and peak at ZT22.2 compared with 0.47 and ZT17.5 in wild-type). Under DEX treatment, both mutant and wild-type exhibit strong rhythmic glutamine concentrations and the phase difference is reduced (model IX, Fig 3E, S5C Fig, S2 Table). Remarkably, glutamine is the only compound of the set which shows such a rescue of both overall levels and circadian rhythmicity by DEX treatment.

### Circadian glutamine metabolism is impaired in *rx3 strong* mutants

Glutamine forms part of several pathways enriched in the rescued gene set. For example, it is a required source of nitrogen for purine and pyrimidine synthesis. Interestingly, an entire chain of enzymes downstream of glutamine entry into the purine biosynthesis pathway shows dysregulation in *rx3 strong* mutants, which is rescued by DEX (S6E Fig). These enzymes act upstream of *IMP* (*inosine 5'-monophosphate*) *dehydrogenase 2* (*impdh2*), which has recently been suggested to be involved in the regulation of circadian rhythms of cell proliferation [18]. *impdh2* expression is also dysregulated in the mutants and rescued by DEX. As genes in many other branches of the purine synthesis pathway are equally rescued (S1 and S3 Tables), GCs seem to regulate a large part of the diurnal transcription within this pathway.

**Fig 4:**
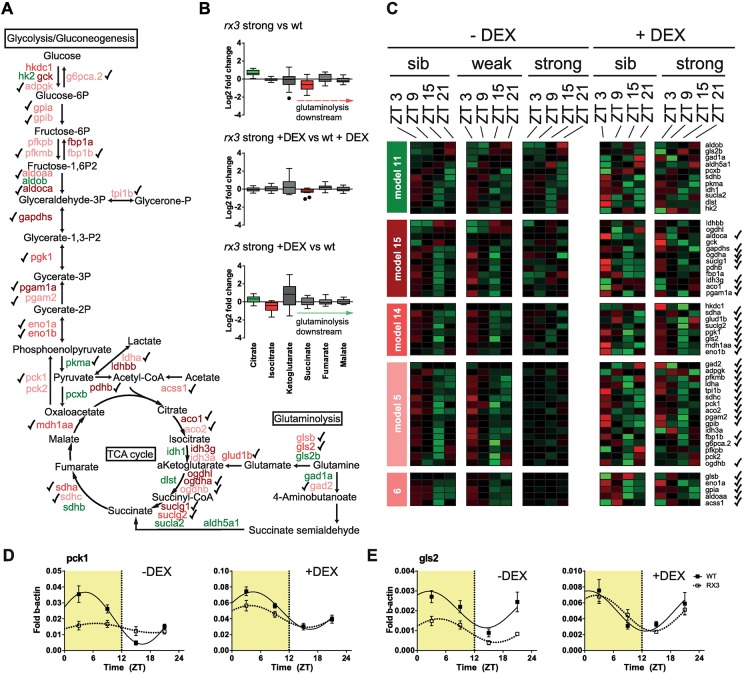
Glucocorticoid dependent alterations of diurnal TCA cycle function. (A) Schematic depicting glycolysis, gluconeogenesis, the TCA cycle, and glutaminolysis, indicating the steps where genes with dysregulated temporal mRNA expression in *rx3 strong* mutants are involved. Gene names are color-coded according to the models in C. Check marks in (A) and (C) indicate rescue of dysregulated expression in *rx3 strong* by DEX treatment. (B) Tukey boxplots showing fold changes of TCA cycle metabolites when comparing the indicated conditions. Statistically significant changes are shown in green for up-regulation and in red for down-regulation. (C) Heatmap of normalized mRNA expression levels of the genes shown in (A), classified according to their expression models. Red, high expression. Green, low expression. (D, E) Gene expression levels of *pck1* (D) and *gls2* (E) mRNA levels in 5 dpf larvae in the indicated conditions. Lines fitted by linear regression if statistical models indicate rhythmicity for wild-type (continuous) and *rx3 strong* mutant samples (broken).

Glutamine is also important for refilling (anaplerosis) of the TCA cycle with α-ketoglutarate when it is deprived of intermediates (Fig 4A). We chose this glutamine-TCA cycle connection for a proof-of-principle analysis. Examination of the cumulated levels of six TCA intermediates shows that citrate levels are higher in the mutants, while succinate levels are reduced (Fig 4B). This finding is consistent with reduced anaplerosis at the level of α-ketoglutarate, leading to an upstream block and downstream depletion of cycle intermediates. In DEX treated conditions the levels are normalized, indicating restored flow. The TCA cycle connects glutaminolysis, glycolysis and gluconeogenesis, and indeed, many TCA cycle and glycolysis enzymes as well as key enzymes of gluconeogenesis show dysregulated expression rescued by DEX treatment (Fig 4A, 4C and 4D). Among them, *phosphoenolpyruvate carboxykinase 1* (*soluble*, *pck1*) removes OAA from the TCA cycle (cataplerosis) and channels it into gluconeogenesis. It has been suggested that this cataplerotic PCK1 function balances anaplerotic refilling of the TCA cycle by glutamine metabolism [19]. Expression of dysregulated genes in this anaplerotic pathway, such as *glutaminase 2 (gls2)*, is also restored by chronic DEX treatment (Fig 4A, 4C and 4E). Therefore, DEX treatment likely restores the cataplerosis/anaplerosis balance of the TCA cycle, thereby normalizing TCA compound and glutamine levels.

### A common feature characterizes regulatory regions of GC-dependent circadian genes

**Fig 5:**
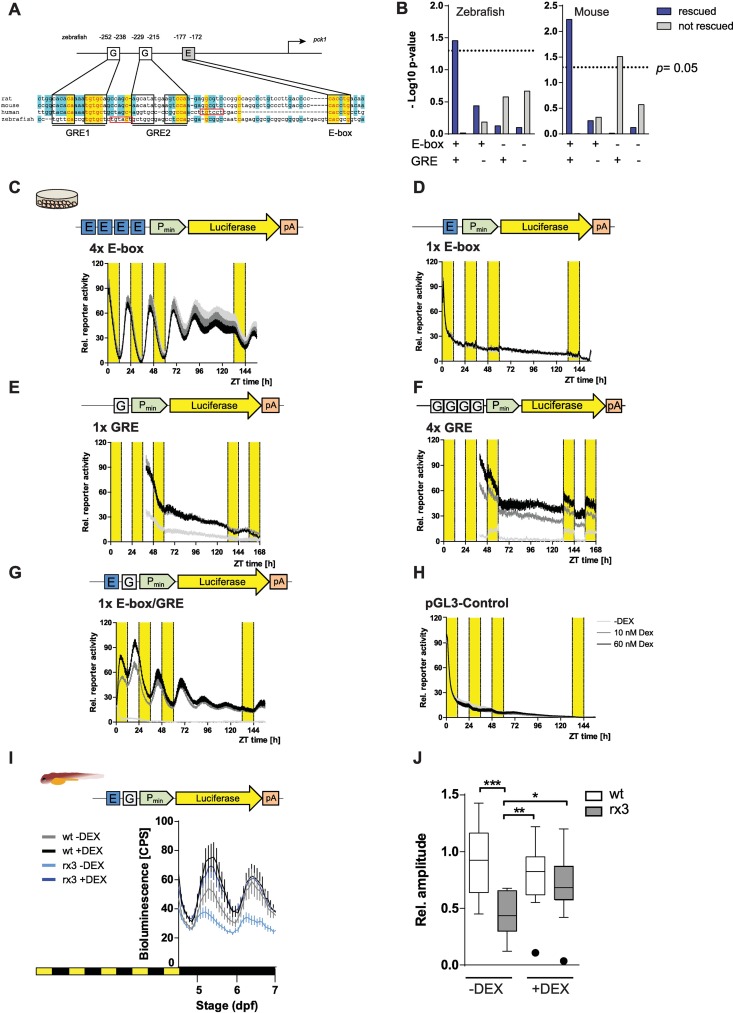
A module of E-box and GRE elements drives circadian expression under constant DEX treatment in *vitro* and *in vivo*. (A) Multiple alignment of the *pck1* promoters of the indicated species. Red boxes indicate GRE half sites. (B) Graph depicting p values for observed vs. random occurrence of E-boxes and GREs in the promoter regions of model 5-6-14-15 genes rescued (green) or not rescued (red) by DEX treatment. (C-H) Bioluminescence traces recorded from zebrafish cells expressing the indicated reporter constructs under different doses of DEX. G, GRE. E, E-box. P_min_,TATA box. pA, polyadenylation site. (I) *In vivo* bioluminescence traces recorded from transgenic zebrafish larvae carrying the 1xE-box/GRE reporter in the indicated phenotype/treatment combinations. (J) Boxplots depicting amplitudes of the bioluminescence oscillations shown in D. *, *p* < 0.05, **, *p* < 0.01, ***, *p* < 0.001.

PCK1 has long been described as a GC target gene and was reported to show circadian expression in mammals [5]. Interestingly, there are two Glucocorticoid Response Elements (GREs) and an E-box in the zebrafish *pck1* promoter region, which are conserved across evolution (Fig 5A). Our RNA-seq study identified *gls2* as another GC inducible circadian gene, and it equally contains both GREs and E-boxes. To test whether this is a typical feature of GC regulated circadian genes, the putative promoter sequences (-1000 +500bp) of the model 5-6-14-15 genes were examined for concomitant presence of both E-box and GRE elements. We observed an enriched co-occurrence of E-boxes and GREs in the rescued genes (hypergeometric test, *p* = 0.04), while no significant enrichment was seen in the non-rescued ones (*p* = 0.97; Fig 5B). Importantly, this enrichment is also observed in the mouse orthologues of the zebrafish genes, indicating evolutionary conservation of this regulatory module. These findings indicate that E-box/GRE modules, which allow for direct transcriptional regulation by both the circadian clock and GCs, are a characteristic feature of GC-dependent diurnal genes.

### A simple E-box and GRE module mediates GC driven gene expression

Can a simple combination of GREs and E-box enhancer elements drive GC dependent circadian patterns of gene expression? Exploiting the direct light sensitivity of zebrafish cells [20], we transfected cells with luciferase reporter constructs driven by different combinations of E-boxes and GREs and exposed them to light-dark cycles at different concentrations of DEX in otherwise GC-depleted culture medium (Fig 5C–5H). Of all combinations, only the E-box/GRE combination (Fig 5G) drove GC dependent circadian transcription, while the other constructs were not sensitive to DEX (1x E-box, 4x E-box) or did not show rhythmic expression (1x E-box, 1x GRE, 4x GRE). Thus, a synergistic interaction between a single E-box and a GRE is sufficient to drive GC-dependent circadian gene expression.

To begin to explore mechanistic aspects of this interaction between E-Boxes and GREs, we examined the bioluminescence patterns driven by the combined E-box/GRE element upon entrainment by different light intensities of the light part of the LD cycle and upon changing distances between the two elements. Interestingly, at the highest light intensity examined, oscillation behaviour was less pronounced (S7A and S7B Fig). Two-way ANOVA analysis indicated that there was no interaction between GC dose (10–60 nM) and light intensity effects (150–1500 lux, S7B Fig). Examination of constructs with different E-box/GRE spacings revealed a slightly less robust oscillation behaviour when the two elements were separated by 10 bps, as in the original construct, than at the other distances (5, 15 and 20 bp; S7C and S7D Fig). These results indicate that the E-box/GRE module is sensitive to light conditions and that interaction between the two elements might be hampered when they are placed relatively nearby on the same side of the DNA helix.

To examine whether the interaction between E-boxes and GREs also occurs *in vivo*, we generated larvae carrying stable genomic insertions of the 1xE-box/GRE reporter. Both in wild-type siblings and in *rx3 strong* mutants, the construct drove rhythmic expression (Fig 5I), indicating that the low levels of cortisol present in the mutants [15] are still sufficient for activation of the construct. Importantly however, the amplitude of expression was clearly reduced in the mutants (Fig 5J). Strikingly, the amplitude difference between mutants and wild-type siblings disappeared when the larvae were treated with DEX during the experiment (Fig 5I and 5J). Thus, E-box/GRE driven bioluminescence rhythms mimic the rhythmicity behavior of many metabolic genes from the RNA-Seq analysis (Fig 1F), suggesting that the E-box-GRE combinations enriched in the GC-dependent diurnal gene set mediate highly efficient regulatory inputs for this expression pattern.

## Discussion

Our data reveal a strong impact of GCs on diurnal gene transcription and key metabolite levels. About 44% of all genes with diurnal expression patterns are dysregulated in GC deficient larvae, and almost half of these can be rescued by constant chronic DEX treatment. This result shows that a surprisingly large part of the GC-dependent diurnal transcriptome does not depend on the diurnal pattern of GC levels themselves. Thus, GCs may have a permissive rather than instructive role in the diurnal expression of these genes. This mechanism of regulation may prevent these genes from being inappropriately regulated by irregular GC rises, such as those during acute stress responses. Recent studies have described direct interactions of circadian clock factors with GRs, leading to circadian modulation of GC signaling [21,22,23]. For example, circadian *Pck1* expression was attributed to direct inhibitory regulation of the GR by CRY binding [22]. Interestingly, the *Pck1* promoter also contains an E-box element which is conserved between fish and mammals. Our *in vivo* reporter gene analysis shows that a simple E-box-GRE combination is sufficient to mediate GC dependent circadian luciferase expression. However, we did not observe circadian regulation of reporter expression driven by four concatemerized GREs. Endogenous Cry levels in zebrafish fibroblasts may not be high enough to mediate transcriptional repression of the 4xGRE reporter *in vivo*, in contrast to the elevated CRY levels upon overexpression used in mouse fibroblasts studies reported previously [22]. Remarkably, GR binding to the GRE in the mouse *Pck1* gene is highest at the time when CRYs reach peak levels and reduced in CRY double knock-out mice [22]. Stable CRY-GR interactions may occur at the promoter itself, where the Clock-Bmal1 bound to the E-box may increase the local concentration of CRY proteins and thereby facilitate their interactions with GRs binding a neighbouring GRE. The increased co-occurrence of E-boxes and GREs in the genes rescued by constant DEX treatment argues in favor of such a mechanism. Of note, zebrafish Cry1a has been implicated in mediating a negative regulatory influence of light on the circadian clock by its interaction with the Clock-Bmal dimer [24], a function that may underlie our observation of reduced oscillations upon exposure to light-dark cycles with higher light intensity. Furthermore, our data indicate that interactions between the GR and the clock machinery may be hampered when the two elements are located nearby on the same side of the DNA helix, as elements separated by 10 bp show slightly less robust oscillations than those separated by 5 bp or 15 bp. Further studies targeted at identifying the full set of factors involved in regulation of transcription by the variants of the element will likely reveal more mechanistic details of the interactions between GCs and the clock in transcriptional regulation.

While the constant DEX treatment clearly influenced gene expression and metabolite patterns, it did not lead to any visible perturbation of the larval phenotype, nor did it affect circadian rhythms of S-phase in the wild-type [15]. Effects of prolonged DEX treatment may appear only at later developmental stages or in adulthood, and only be noticeable upon challenges to the organism. This is seen in humans or other mammals exposed to high DEX levels during development, which later in life show increased disease susceptibility and altered responses to stress [25,26]. The DEX-treatment related changes to metabolites or gene expression patterns revealed within our data set may provide interesting starting points for understanding the mechanistic principles of such long-term effects.

Constant DEX treatment rescues circadian rhythms of S-phase in *rx3 strong* mutants [15]. Our transcriptome analysis reveals that a number of cell cycle genes, related to all phases of the cell cycle, show a similar pattern, as do numerous genes acting in metabolic pathways implicated in the regulation of cell proliferation. Surprisingly, fewer cell cycle genes than metabolic genes were affected by the loss of GCs. Also, more of the affected metabolic genes were rescued by constant DEX treatment. This indicates that GC dependent metabolic control may play a more important role in circadian cell cycle rhythms than GC regulation of cell cycle genes. Strikingly, of the metabolites examined, only glutamine showed a restoration of both overall levels and rhythmicity by constant DEX treatment. Glutamine plays a major role in cell proliferation related pathways such as purine synthesis or anaplerosis of the TCA cycle. Thus, it emerges as a potential key metabolite in the circadian orchestration of cell proliferation. The presence of a circadian rhythm of glutamine levels in human blood [27] and in rat liver [28] suggests that such functions are conserved across evolution.

Intriguingly, we observed a strong accumulation of glyoxylate in *rx3 strong* mutants, which was prevented by DEX treatment. This glyoxylate accumulation may reflect the bypassing of part of the disturbed TCA cycle by the so-called glyoxylate cycle. However, apart from *C*. *elegans*, the presence of this cycle in metazoans is controversial, and orthologues of the glyoxylate cycle enzymes *isocitrate dehydrogenase* and *malate synthase* are reported to be absent or pseudogenes in vertebrates [29]. Interestingly, the zebrafish genome contains a potentially functional *malate synthase*-like sequence (*ENSDARG00000074684*), which showed DEX rescue of a dysregulated expression pattern in *rx3 strong* mutants (S1 Table). Also peroxisomal and mitochondrial glyoxylate detoxification is dysregulated in the mutants, involving decreases in expression of glyoxylate metabolizing enzymes and increases in glyoxylate producing enzymes. Importantly, three of the dysregulated genes encode enzymes linked with human genetic disorders of glyoxylate detoxification (*alanin-glyoxylate amino transferase* [*agxtb*], *glyoxylate reductase/hydroxypyruvate reductase* [*grhprb*] and *4-hydroxy-2-oxoglutarate aldolase 1* [*hoga1*], which are involved in human primary hyperoxalurias type I-III, respectively [17]). With one exception (*grhprb*), constant DEX rescues expression patterns of all these genes. Thus, GCs may be a promising candidate for treatment of patients in which insufficient amounts of functional protein are produced [17,30].

Another intriguing finding is our observation that the aberrant accumulation of both BCAA and aromatic AA levels in the mutants could not be restored by DEX treatment. Dysregulation of these metabolites may reflect a perturbation of other regulatory inputs in addition to GCs in the secondary AI model. Alternatively, our temporally constant GC replacement by DEX may not be sufficient for proper functional restoration of all pathways influencing BCAA and AAA levels. It will be interesting to explore if other treatment schemes are more efficient in restoring BCAA and AAA levels, and whether these amino acids also show disturbed regulation in human patients.

In summary, our study establishes zebrafish larvae as an easily accessible model for studies targeting metabolic aspects of GC related disease and GC therapy. In addition, our work reveals a massive impact of GCs on the diurnal patterns of gene transcription. Surprisingly, a large part of this diurnal regulation does not require changing levels of GCs themselves, and we provide a model based on simple enhancer element interactions that can explain this behavior.

## Materials and Methods

### Ethics statement

Animal experiments were conducted in accordance with German animal protection regulations and approved by local regulatory authorities (Regierungspräsidium Karlsruhe, approval number Aktenzeichen 35–9185.81/G-83/14 and 35–9185.81/G-242/15).

### Zebrafish husbandry

Fish (AB wild-type and the mutant lines *rx3*^*t25327/t25327*^ [*rx3 strong*] and *rx3*^*t25181/t25181*^ [*rx3 weak*] [14]) were raised and bred as described [31].

### Total RNA extraction, cDNA synthesis and quantitative real-time PCR

Total RNA extraction, cDNA synthesis and qPCR were carried out as described [32]. Primer sequences used were: β-actin: fw: 5’-gcctgacggacaggtcat-3, rv:’ 5’-accgcaagattccataccc-3’; apoa1: fw: 5’-cttgacaacctggacggaac-3, rv: 5’-gcatattcctggagcttggt-3; arntl1a: fw: 5’-tagagcgctgtttgctgatg-3’, rv:5’-gacccgtggacttcagtgac-3; cyp3a: fw: 5’-ccaaagacaacacgaagcag-3’, rv: 5’-acaagatctcgtggtcactcag-3’; rbp4: fw: 5’-ccgaagatccagctaagttca-3, rv:’ 5’-caatgatccagtggtcgtca-3’; si:dkey-18a10.3: fw: 5’-ctttgtgcgccaactcaac-3’, rv: 5’-tttaggcaagccggagtcta-3’; pck1: fw: 5’-tgacgtcctggaagaacca-3’, rv: 5’-gcgtacagaagcgggagtt-3’; gls2a: fw:5’-gacatgacagcagctcttgact-3’, rv: 5’-tgcctgactcacatgtcacc-3’.

### Whole mount in situ hybridization (WISH)

Synthesis of probes against *lhx4*, *apoa1*, and *zgc*:*158293* and whole-mount in situ hybridization was carried out following Armant et al. [33].

### Treatment and sampling

1 dpf *rx3* mutant and wildtype sibling embryos from pooled clutches were separated and transferred into cell culture flasks containing E3 medium (5 mM NaCl, 0.17 mM KCl, 0.33 mM CaCl_2_, 0.33 mM MgSO_4_, 0.1% methylene blue). For GC treatment, the medium was supplemented with either 25 μM Dexamethasone (DEX) solved in 0.1% DMSO or 0.1% DMSO alone as a control. Embryos/larvae were kept at 28°C in an incubator under a 12 h light:12 h dark (LD) exposure. Larvae were sampled in liquid nitrogen at the indicated *Zeitgeber* times (ZT, ZT3 = 3 hours after lights on) starting at 5 dpf and stored at -80°C until further processing.

### ^1^H-NMR spectroscopy

To ensure efficient and reproducible sample preparation for NMR metabolomics of zebrafish larvae, we have established an extraction protocol that generates highly reproducible data. 50 larvae per sample were collected in homogenization tubes (PeqLab, #91-PCS-CK14), snap-frozen in liquid nitrogen and lyophilized overnight to avoid recovery of enzymatic activity. Lyophilized samples were stored at -80°C for a maximum of 2 days. For extraction, 1 ml of acetonitrile/water (1:1) and ceramic beads (#91-PCS-CK14, PeqLab) were added to the larvae and extracted with a liquid nitrogen cooled cell shaker (Precellys 24, PeqLab) according to the manufacturer’s instructions, with the following settings: 6,000 rpm, 4x20 s, 120 s. Homogenates were vortexed for 1 min and incubated on ice for 10 min. Next, the samples were transferred into fresh vials, and were briefly centrifuged at 4°C to remove debris. 750 μl of supernatant were transferred into fresh vials, and 620 μl of ultrapure water (HPLC grade) were added. Samples were vortexed before lyophilization overnight. For measurements, 650 μL of D_2_O/buffer (1.5 M KH_2_PO_4_, 2 mM NaN_3_, 0.1% (v/v) TSP (= 3,3-(trimethylsilyl)-2,2',3,3'-tetradeuteropropionic acid) in D_2_O) (9:1) were added to the extracts, and 600 μl of this mixture were transferred into a 5 mm standard NMR tube. Spectra were recorded on a Bruker Avance III 600 spectrometer equipped with a ^1^H,^13^C,^15^N-TCI triple resonance cryoprobe. 1D spectra were recorded with 64k data points, 90.5 receiver gain and 32 scans for comparing the phenotypes and 64 scans for comparing the DEX treatment at 300 K using a 1D NOESY experiment with presaturation for water suppression. A mixing time of 10 ms and a prescan delay of 10 s were used. Pulse length was determined automatically by the Bruker AU program pulsecal and presaturation was set corresponding to a 25 Hz pulse. Irradiation frequency for water suppression was optimized prior to acquisition. Spectra were processed identically with an exponential apodization function with line broadening of 0.3 Hz. Automatic phasing, baseline correction and referencing were done by the Bruker AU program apk0.noe. Additionally, J-resolved spectra were recorded with identical pulse lengths, presaturation, irradiation frequency and receiver gain as the corresponding 1D NOESY. Spectra were recorded with 8192 data points in the direct dimension and 40 increments in the indirect dimension. One scan was acquired per increment. Spectra were processed with 16k × 256 data points and a sine window function in both dimensions.

### Determination of metabolite levels via UPLC-FCS and IC-CD

We employed ultra-performance liquid chromatography with fluorescence detection (UPLC-FLR) for targeted quantification of amino acids and ketoacids (S8 Fig) and ion chromatography with conductivity detection (IC-CD) for quantification of other organic acids. 30 larvae per sample (in triplicates) were collected for absolute quantification of amino acids and α-ketoacids (glyoxylate) and for organic acid content each. For extraction of free amino acids and α-ketoacids 300μl 0.1M HCl was used. Derivatization and separation of amino acids was performed as described by Yang et al. [34]. For derivatization of α-ketoacids 150 μl of the acidic extract was mixed with an equal volume of 25 mM OPD (o-phenylendiamine) solution and incubated at 50°C for 30 min. The derivatized α-ketoacids were separated using an Acquity HSS T3 column (100 mm x 2.1 mm, 1.7 μm, Waters) connected to an Acquity H-class UPLC system. Prior separation, the column was heated to 40°C and equilibrated with solvent A (0.1% formic acid in 10% acetonitrile) at a flow rate of 0.55 ml/min. Separation of α-ketoacid derivates was achieved by increasing the concentration of solvent B (acetonitrile) in solvent A as follows: 2 min 2% B, 5 min 18% B, 5.2 min 22% B, 9 min 40% B, 9.1min 80% B and hold for 2min, and return to 2% B in 2 min. The separated derivates were detected by fluorescence (Acquity FLR detector, Waters, excitation: 350 nm, emission: 410 nm) and quantified using ultrapure standards (Sigma). For quantification, a linear seven-point calibration curve ranging from 0.3–15 pmol on column was used (R^2^ > 0.99). Data acquisition and processing was performed with the Empower3 software suite (Waters). Organic acids were extracted with 700 μl ultra-pure water for 20 min at 95°C. These compounds were separated using an IonPac AS11-HC (2mm, ThermoScientific) column connected to an ICS-3000 system (Dionex) and quantified by conductivity detection after cation suppression (ASRS-300 2mm, suppressor current 95–120 mA). Prior separation, the column was heated to 30°C and equilibrated with 5 column volumes of solvent A (ultra-pure water) at a flow rate of 0.38 ml/min. Separation of anions and organic acids was achieved by increasing the concentration of solvent B (methanol) and solvent C (100mM NaOH) in buffer A as follows: 8 min 4% C, 11 min 10% C, 18.2 min 20% B / 18.1% C, 27.5 min 20% B / 21% C, 32 min 24% C, 43 min 30% C, 47 min 40% C, 48 min 90% C for 8 min, and return to 4% C in 9 min. A linear three-point calibration curve was used for quantification of organic acids (0.5–5nmol on column; R^2^ > 0.99) Data acquisition and processing was performed with the Chromeleon 6.7 software (Dionex). Rhythmic properties of metabolite levels were assessed with the model selection based method described in Atger et al. [16] (see also below, **Rhythmicity assessment in different genotypic backgrounds**).

### Transcriptomic studies

Library preparation and sequencing were performed by the IGBMC Microarray and Sequencing Platform and by the Next Generation Sequencing and Genomics facility of the BioInterfaces research programme at KIT. RNA integrity numbers measured with a 2100 Bioanalyzer (Agilent) were 10 for all samples. cDNA libraries were generated using the directional mRNA-seq sample preparation kit (#15018460, Rev.A, October 2010, Illumina). Single-end 54 nt reads were obtained with a Genome Analyzer IIx.

### Data analysis

#### Identification and quantification of compounds by NMR spectroscopy

Individual compounds from zebrafish extracts were identified using both NMR chemical shift databases and spiking of samples. All compounds mentioned in the article were identified using the BBIOREFCODE databank from Bruker (Bruker BioSpin AG, Rheinstetten, Germany) or the program CHENOMX with inherent database (Chenomx Inc., Edmonton, Canada). For every compound reported we additionally added small amounts of pure substances to the zebrafish extracts and re-recorded 1D ^1^H-NMR spectra. In S9 Fig, corresponding spectra for zebrafish extracts with and without addition of glutamine (A, turquoise), creatine (B, orange), arginine (C, purple), glutamate (C, green), carnitine (D, blue), lactate (D, light green) and alanine (D, red) are shown overlaid.

NMR spectra were displayed and analysed by principal component analysis (PCA) using AMIX 3.9.11. Spectra were manually divided into buckets. Bucket intensities were divided by bucket width, normalized to total intensity and not scaled. Quantification of buckets was achieved by summation of intensities within buckets. For the PCA analysing the untreated phenotypes (Fig 1C, S1A Fig), 46 spectra were divided into 116 buckets. For the PCA analysing the DEX treatment (Fig 1D, S1B Fig), 60 spectra were divided into 145 buckets. Corresponding bucket tables are given in the supplementary information (S4 Table).

#### RNA-Seq data processing and analysis

Image analysis and base calling were performed using the Illumina pipeline (Real Time Analysis and Offline Base Caller 1.8). All raw sequence data are available from the GEO database (accession number GSE76073, http://www.ncbi.nlm.nih.gov/geo/). Reads were mapped onto the Zv9 assembly of the zebrafish genome using STAR 2.3.8 [35]. Uniquely mapped reads were counted for each gene locus as annotated in Ensembl v75 using the Python Package HTseq v0.5.3p1. Read counts were normalized using DESeq2 [36]. For downstream analysis a variance stabilizing transformation was applied to the normalized data [37].

#### Rhythmicity assessment in different genotypic backgrounds

The rhythmicity in different conditions of gene expression was assessed as described in Atger et al. [16]. Briefly, this method is based on multiple linear regression and a subsequent model selection using the Bayesian information criterion (BIC). To this end, we first defined the relation y(t) = μ + αcos((2π/24 h)t) + βsin((2π/24 h)t) + noise (y is the log2 transformed signal, μ is mean, t is *Zeitgeber* time, α and β are the coefficients of the cosine and sine functions, respectively). In order to compare rhythmicity across the different conditions (e.g. phenotype and treatment), we generated different models. For two (WT+DEX and strong+DEX) or three conditions (WT, weak, strong), we defined models with α and β to be either zero (non-rhythmic pattern) or non-zero (rhythmic pattern) within the different conditions. In addition, α and β can be shared within any combination of the two or three conditions. These coefficients determine the peak-trough amplitude ($2\sqrt{\alpha^{2}+\beta^{2}}$) and the phase [Arctan ($\frac{\beta }{\alpha }$)]. For example, a gene could be non-rhythmic or rhythmic in a certain condition. Rhythmic conditions may share the phase and amplitude with any other condition. For example, there are 5 and 15 resulting models for two and three conditions, respectively. We use linear regression to solve each model and model complexity is then controlled by applying a BIC based model selection [38], with $BIC=nln\left(\frac{RSS}{n}\right)+kln(n)$ (RSS is the residual sum of squares of the linear regression, n is the number of time points, and k is the number of parameters). Schwarz weight (w_j_) is used to assess the confidence of a model: $w_{j}=\frac{e^{\frac{1}{2}\Delta {BIC}_{j}}}{\sum_{M}e^{\frac{1}{2}\Delta {BIC}_{m}}}$, with Δ*BIC*_*j*_ = *BIC*_*j*_ − *BIC*_m*_ (m*is the minimum BIC value in the set of models). We interpreted w_j_ as the confidence for model j being the optimal model as defined by the BIC framework within the set of defined models. Genes were categorized as “ambiguous” if the BIC weight (BICW) did not reach a threshold chosen according to the number of conditions examined (0.3–0.5). Relative amplitude of gene expression was approximated as the difference of maximum and minimum expression levels normalized by the mean expression at all time points.

Rhythmicity of metabolite data was assessed by the same approach with some modifications. We added a non-periodic time-dependent coefficient to the harmonic regression in order to match the overall decline observed in some metabolites. For the four conditions (WT -DEX, *rx3* strong–DEX, WT + DEX, and *rx3* strong + DEX) we identified 9 models (I-IX) that gave the best match to the data (described in S2 Table).

#### Gene ontology (GO) and metabolic term enrichment

Enrichment analyses were performed using the Bioconductor package TopGO [39]. Biological Processes (BP_all) and the KEGG pathway annotation were included in the analysis. In addition, a manually curated set of zebrafish genes linked with important metabolic pathways assembled from KEGG and Ensembl annotations (S3 Table) complemented the approach. Statistically significant overrepresentations were determined by a hypergeometric test. Multiple testing was corrected by FDR correction [40]. Only categories with a p-value smaller than 0.05 were considered as significantly enriched.

#### Transcription factor binding site analysis

To identify enriched E-box and GRE transcription factor binding sites (TFBS) in the putative promoter sequences of the indicated gene sets, the region 1500 bp upstream and 500 bp downstream of the transcription start site was retrieved with a custom R script. The TFBS motifs were localized in these regions using FIMO [41], employing positional weight matrices (PWMs) as previously defined (E-box: [42]; GRE: [43]). Hits with a *p*-value lower than 2 x 10^−4^ were retained. Statistical significance of GRE and E-box overrepresentation was determined by hypergeometric testing. The putative *pck1* promoters of different species were manually aligned.

### Cell culture maintenance and bioluminescence reporter assays

Zebrafish cells were maintained as described [44] in Leibowitz’s (L-15) medium (Life Technologies, #11415–049) supplemented with antibiotics and 15% (PAC2 cells) or 17% (AB.9 GRE:Luc cells) Fetal bovine serum (%[v/v], FBS, Biochrom AG, #S0115). Bioluminescence studies for cells and larvae were carried out as reported previously [32,44,45].

#### Bioluminescence studies in cell culture

All transfected plasmids were derivatives of the pT2Luci:MCS plasmids [44]. *cis*-regulatory elements were inserted into this plasmid using 5’ phosphate modified oligonucleotides (Life Technologies). Oligonucleotides (1 nmol each) were assembled in a 6 mM Tris-HCl buffer (pH 7.5) supplemented with 6 mM MgCl_2_, 10 mM NaCl_2_, and 1 mM DDT in a total volume of 100 μl. The mix was heated up to 95°C for 5 min and then gradually cooled down to room temperature over a period of 3 h to allow proper oligonucleotide annealing. Oligonucleotides sequences were: 1x E-box: fw: 5’-agctgcacgtgtactcggaagc-3’, rv: 5’-gatcgcttccgagtacacgtgc-3’; 1x GRE: fw: 5’-agctgggtacattttgttcttactcggaagc-3’, rv: 5’-gatcgcttccgagtaagaacaaaatgtaccc-3’; 1x E-box/GRE (= 1x E-box/GRE (10)): fw: 5’-agctgcacgtgtactcggaagggtacattttgttctc-3’, rv: 5’-gatcgagaacaaaatgtacccttccgagtacacatgc-3’; 4x GRE: fw: 5’-tcgatggtacattttgttctagaacaaaatgtaccggtacattttgttctggtacattttgttcta-3’, rv: 5’-tcgatagaacaaaatgtaccagaacaaaatgtaccggtacattttgttctagaacaaaatgtacca-3’. 1xE-Box/GRE (5): fw: 5’- tcgatcacgtgtactcggtacattttgttcta-3’, rv: 5’-tcgatagaacaaaatgtaccgagtacacgtga-3’; 1xE-Box/GRE (15): fw: 5’- tcgatcacgtgtactcggaagtactcggtacattttgttcta-3’, rv: 5’- tcgatagaacaaaatgtaccgagtacttccgagtacacgtga-3’; 1xE-Box/GRE (20): fw: 5’- tcgatcacgtgtactcggaagtactcggaagggtacattttgttcta-3’, rv: 5’- tcgatagaacaaaatgtacccttccgagtacttccgagtacacgtga-3’. The generation of the 4x E-box construct has been reported previously [32].

The cell culture bioluminescence studies were carried out with stably transfected 4x GRE cell lines [44] or with cells transiently transfected with the indicated constructs. Transfection of PAC2 cells was performed in 6-well plates using FuGENE HD (Promega) following the instructions of the manufacturer. 900 ng pT2Luci:MCS derivatives were transfected together with 100 ng pCS-TP [46] for tol2 transposase facilitated integration into the genome. The constitutively active pGL3-Control vector (Promega) was used as a control. 35,000 cells were seeded into white 96-well plates (PerkinElmer, #6005299) and then kept overnight at 28°C. The next day, maintenance medium was replaced by L-15 medium without phenol red containing 0.5 mM luciferin (Promega, #E1603) and 15% or 17% (v/v) charcoal treated FCS (Biochrome) for PAC2 and AB.9 cells, respectively. Cells were treated with different concentrations of DEX (10 nM, 60 nM) or DMSO as a control before reporter activity was determined for several days at 28°C under the indicated lighting conditions with an EnvisionXCite Plate Reader (PerkinElmer). For light intensity experiments, cells were treated with DEX (10 nM, 60 nM) or DMSO and subsequently entrained under the indicated light intensities and light dark periods before bioluminescence was measured in constant darkness.

#### *In vivo* bioluminescence measurements in zebrafish larvae

To obtain a stable E-GRE::Luc transgenic zebrafish line in the *rx3 strong* mutant background, embryos from an incross of *rx3 strong* heterozygous carriers were injected with the pT2Luci: 1x E-box/GRE plasmid and Tol2 transposase mRNA. Embryos/larvae were exposed to LD for 5 days, then screened for rhythmic luciferase activity recorded from single larvae in constant darkness (DD) as described [32]. Positive larvae were raised and outcrossed with *rx3 strong* heterozygous carriers in order to identify founders and obtain the combined mutant and transgenic F1 generation. DEX (10 μM) and DMSO treatment of the larvae was carried out as described [44].

### Statistical analysis

In case of multiple comparisons, *p*-values were adjusted using the Benjamini-Hochberg method [40].

## Supporting Information

S1 Fig Quality assessment, validation and analysis of RNA-Seq data. (A) Bar diagram illustrating the mapping statistics of the indicated samples. “Multiple loci” indicates reads that map to up to 10 loci, “too many loci” refers to reads that map to more than 10 loci. (B) Quantitative measurements of mRNA expression for *cypa3*, *rbp4*, *si*:*dkey-18a10*.*3*, *apoa1*, and *arntl1a* (*bmal1a*) by RT-qPCR (x-axis) highly correlate with the corresponding RNA-Seq data (y-axis). The Spearman’s correlation coefficient for the values determined by the two techniques is 0.8697 (*p*-value <0.0001). (C) The mutant specific alterations in gene expression as determined by the RNA-Seq study for *zgc*:*158291*, *lhx4*, and *apoa1* are detectable in 24 hpf embryos using whole-mount *in situ* hybridization. *zgc*:*158291* is expressed in cranial ganglia (anterodorsal lateral line ganglia (gAD) and statoacoustic ganglion(gVIII)) across all phenotypes (siblings, *rx3 weak*, and *rx3 strong*). The expression domain in the optic fissure (of) is lacking in both blind mutants. *lhx4* is highly expressed in wild-type siblings in the pineal (p), the trigeminal ganglion (gV) and the adenohypophyseal placode (ap). The expression of *lhx4* is affected in a gradual manner: *rx3 weak* mutants still exhibit a weak expression in the presumptive adenohypophysis. *rx3 strong* mutants lack the expression domain completely. *apoa1* expression in the subpallial telencephalon (sp) shows a broad expansion exclusively in *rx3 strong* mutants. (D,E) Biological functions enriched in model 11 genes (D) and model 5-6-14-15 genes (E). (F,G) Metabolic pathways enriched among model 11 genes (F) and model 5-6-14-15 genes (G).
(TIF)

S2 Fig Overview of rhythmic patterns in all models and of phase and amplitude values in “affected” models. **(**A-C) Schematic overview of rhythmic patterns of all models. Black curved line: oscillation with same phase and amplitude as wild-type, black straight line: no oscillation, red or green curved lines: oscillation with a phase or amplitude different from wild-type. The green curved line indicates an oscillation with an amplitude and/or phase that is both different from wild-type (black) and from the other changed oscillation (red). (A) Untreated transcriptome models, (B) DEX treated transcriptome models, (C) metabolite models. (D-E). Violin plots of amplitudes across all genes belonging to model 14 (D), model 15 (E), model 5 (F) and model 6 (G), comparing all phenotypes still showing rhythmicity. (H-K) Phase distribution of wild-type and *rx3 weak* (left) or wild-type and *rx3 strong* (right) for model 14 (H), model 15 (I), model 5 (J) and model 6 (K). “no rhythmicity” = no comparison can be made, as the *rx3 strong* phenotype is arrhythmic.
(TIF)

S3 Fig Expression patterns of circadian clock and cell cycle related genes. Heatmaps depicting mRNA levels of circadian clock genes (A) and cell cycle genes (B) in samples of the indicated conditions and time points. Genes are sorted according to their classifications within models (11, 5, 6, 14, 15). Expression values are colour-coded with green and red for low and high relative expression levels, respectively. Expression levels are normalized by the mean of each gene within each treatment/phenotype combination. Check marks indicate genes whose deregulated expression in *rx3 strong* mutants is rescued by DEX treatment.
(TIF)

S4 Fig Exemplary gene expression profiles illustrating typical expression patterns of rescued genes. Red curves: *rx3 strong*, green curves: *rx3 weak*, black curves wild-type siblings.
(TIF)

S5 Fig Metabolite patterns in *rx3 strong* mutants with and without DEX treatment. (A,B) Principal component analysis (PCA) loading plots of the NMR spectra of untreated (A) and DEX treated (B) samples. Color-coding indicates metabolites which correspond to the spectral peaks contributing the most to the first three principal components (PC1-3, % indicates contribution to total variance): creatine (purple), glutamine (light green), lactate (yellow), betaine (dark green), glycine (blue). (C) Left: Temporal profile of glutamine levels as determined by NMR analysis in wild-type (black) and *rx3 strong* mutants (white) in the absence or presence of dexamethasone (DEX). Error bars indicate SEM. Right: Tukey boxplot depicting glutamine levels at all examined time points in wild-type siblings (WT, white) and *rx3 strong* mutants (RX3, grey) in the absence or presence of DEX. (D-K) Temporal profiles of the indicated branched chain amino acid (BCAA; D and E), aromatic amino acid (AAA; F and G), ornithine-urea cycle (OUC) amino acid (H, I, J) and lysine levels (K) determined by UPLC-FLR and IC-CD analysis in wild-type (black) and *rx3 strong* mutants (white) in the absence or presence of dexamethasone (DEX). Lines fitted by linear regression shown as continuous if statistical models indicate rhythmicity and as broken if the fit was non-rhythmic. When the model assignment was “ambiguous”, no fit is indicated. Error bars indicate SEM. Right: Tukey boxplots depicting metabolite levels at all examined time points in wild-type siblings (WT, black) and *rx3 strong* mutants (RX3, white) in the absence or presence of DEX.
(TIF)

S6 Fig mRNA expression patterns in selected pathways showing metabolite changes in *rx3 strong* mutants. Left panels: Schematics of metabolism pathways indicating steps where genes with deregulated mRNA levels in *rx3 strong* mutants are involved. Right panels: Heatmaps of normalized gene expression levels of deregulated genes arranged according to model classification. Expression values are color-coded with green and red for low and high relative expression levels, respectively. Check marks indicate genes whose deregulated expression in *rx3 strong* mutants is rescued by DEX treatment. (A) Phenylalanine and tyrosine metabolism. (B) Valine, leucine, and isoleucine metabolism. (C) Glyoxylate metabolism. HO-Glu, 4-hydroxy-2-oxoglutarate; H-Glu, 4-hydroxy-glutamate, PyrrOHcarbox, pyrroline-5-carboxylate. Scheme following Salido et al. [17]. (D) Ornithine urea cycle (OUC). (E) Purine metabolism.
(TIF)

S7 Fig Effects of light intensity and spacing between elements on oscillation behaviour of E/GRE module driven reporter gene expression. (A,B) Light intensity effects. (A) Bioluminescence traces recorded from zebrafish cells expressing the 1x E-box/GRE reporter constructs under different concentrations of DEX (10 nM, 60 nM) or DMSO as a control. Cells were entrained with 12 h light (yellow) / 12 h dark (black) cycles using different light intensities (150 lux, 400 lux, 1500 lux), then bioluminescence was recorded under constant darkness. (B) Tukey boxplots depicting the relative amplitudes of the first and the second cycle of the monitored bioluminescence oscillations shown in A. Relative amplitude is lower at the highest light intensity. Two-way ANOVA indicates significant effects on amplitude for light, but not for DEX treatment or an interaction between the two treatments. (C,D) Element spacing effects: (C) Bioluminescence traces recorded from zebrafish cells expressing the 1x E-box/GRE reporter constructs with different spacing (5 to 20 bp) between the E-box and GRE enhancer elements. (D) Tukey boxplots depicting the relative amplitudes of the first and the second cycle of the monitored bioluminescence oscillations shown in C. *, *p* < 0.05. n.s, not significant.
(TIF)

S8 Fig Examples of UPLC-FLR spectra used for targeted quantification of amino acids and ketoacids. Shown are three examples for (A) glyoxylate, pyruvate, and ketoglutarate in samples at ZT9 (B) serine, glutamine, glycine, and histidine in samples at ZT3 (C) tyrosine, valine, and methionine in samples at ZT21. Black, wild-type; red, *rx3 strong* mutants
(TIF)

S9 Fig Overlay of spectra of zebrafish extracts with the same spectra spiked with pure metabolites to allow unambiguous assignment of the corresponding compounds. The shown spectra are extracts from wildtype siblings (D, E), rx3 strong mutants (C) and wildtype siblings treated with DEX (A, B) at ZT21 (A) or ZT3 (B-D). Extracts without spiked metabolites are always colored black. In some cases (C and D), samples were spiked two and three times with known compounds, as clear identification of the corresponding signals was still possible. Exemplary spectra of zebrafish extract with added glutamine (turquoise), creatine (orange), arginine (purple), glutamate (green), carnitine (blue), lactate (light green) and alanine (red) are shown. As the signals are frequently in crowded areas, spiking is necessary because comparison with databases would be inconclusive. The spiked carnitine signals in this region do not overlap with signals in the pure extract. For clarification, the excerpt of the corresponding J-resolved spectrum with easily identifiable multiplet patterns for carnitine is shown as well (E).
(PNG)

S1 Table RNA-Seq analysis results. (XLS)

S2 Table UPLC-FLR and IC-CD data overview. (XLSX)

S3 Table List of zebrafish metabolic pathway genes (XLSX)

S4 Table Bucket tables for NMR analysis. (XLSX)
