# Supplementary figures and images for: Extensive Regulation of Diurnal Transcription and Metabolism by Glucocorticoids
Source: PLoS Genet. 2016 Dec 12;12(12):e1006512. doi: 10.1371/journal.pgen.1006512 (PMC5191836; doi:10.1371/journal.pgen.1006512)

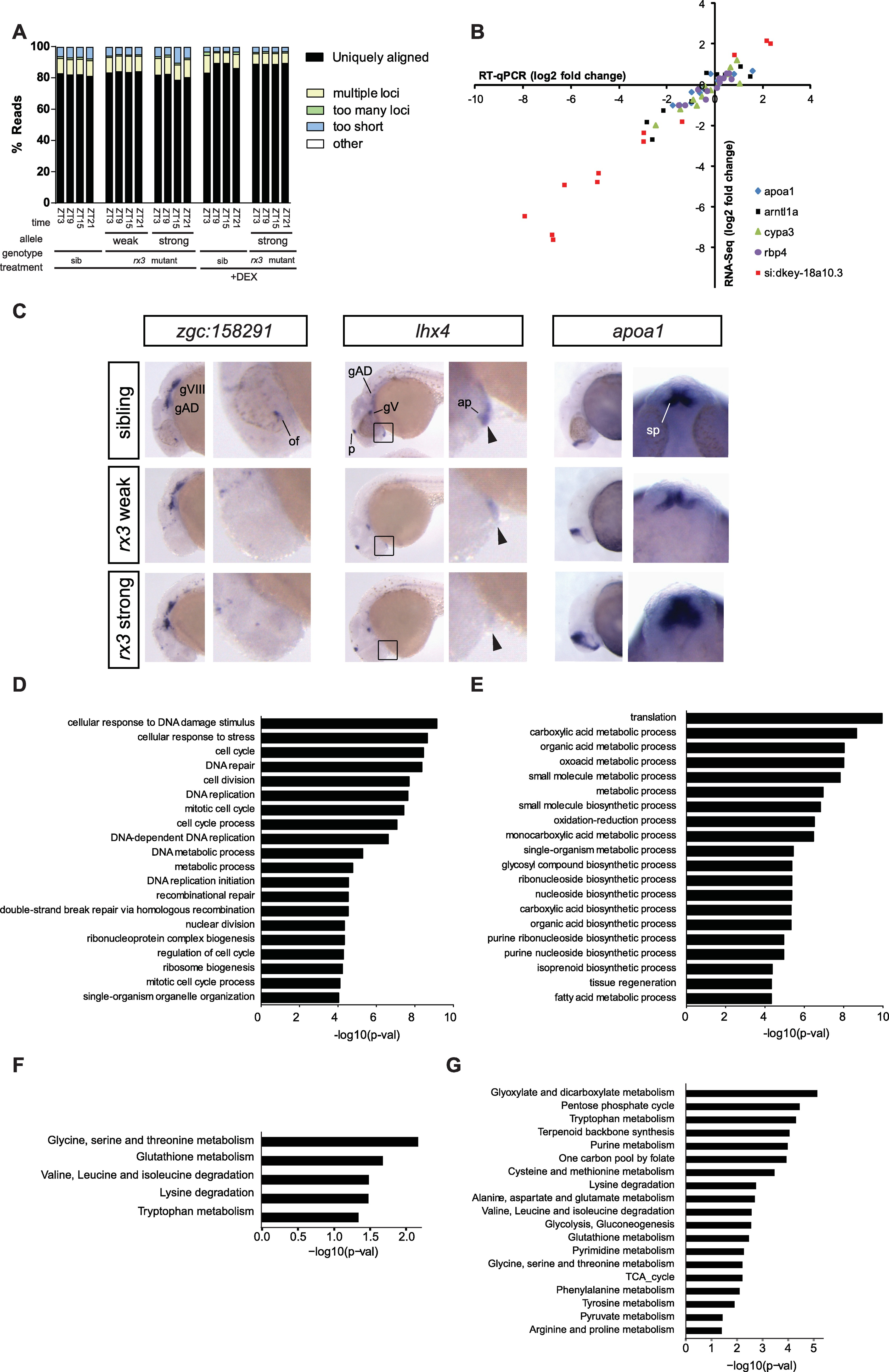

Supplement: S1 Fig — (A) Bar diagram illustrating the mapping statistics of the indicated samples. “Multiple loci” indicates reads that map to up to 10 loci, “too many loci” refers to reads that map to more than 10 loci. (B) Quantitative measurements of mRNA expression for cypa3, rbp4, si:dkey-18a10.3, apoa1, and arntl1a (bmal1a) by RT-qPCR (x-axis) highly correlate with the corresponding RNA-Seq data (y-axis). The Spearman’s correlation coefficient for the values determined by the two techniques is 0.8697 (p-value <0.0001). (C) The mutant specific alterations in gene expression as determined by the RNA-Seq study for zgc:158291, lhx4, and apoa1 are detectable in 24 hpf embryos using whole-mount in situ hybridization. zgc:158291 is expressed in cranial ganglia (anterodorsal lateral line ganglia (gAD) and statoacoustic ganglion(gVIII)) across all phenotypes (siblings, rx3 weak, and rx3 strong). The expression domain in the optic fissure (of) is lacking in both blind mutants. lhx4 is highly expressed in wild-type siblings in the pineal (p), the trigeminal ganglion (gV) and the adenohypophyseal placode (ap). The expression of lhx4 is affected in a gradual manner: rx3 weak mutants still exhibit a weak expression in the presumptive adenohypophysis. rx3 strong mutants lack the expression domain completely. apoa1 expression in the subpallial telencephalon (sp) shows a broad expansion exclusively in rx3 strong mutants. (D,E) Biological functions enriched in model 11 genes (D) and model 5-6-14-15 genes (E). (F,G) Metabolic pathways enriched among model 11 genes (F) and model 5-6-14-15 genes (G). (TIF) [file pgen.1006512.s001.tif]

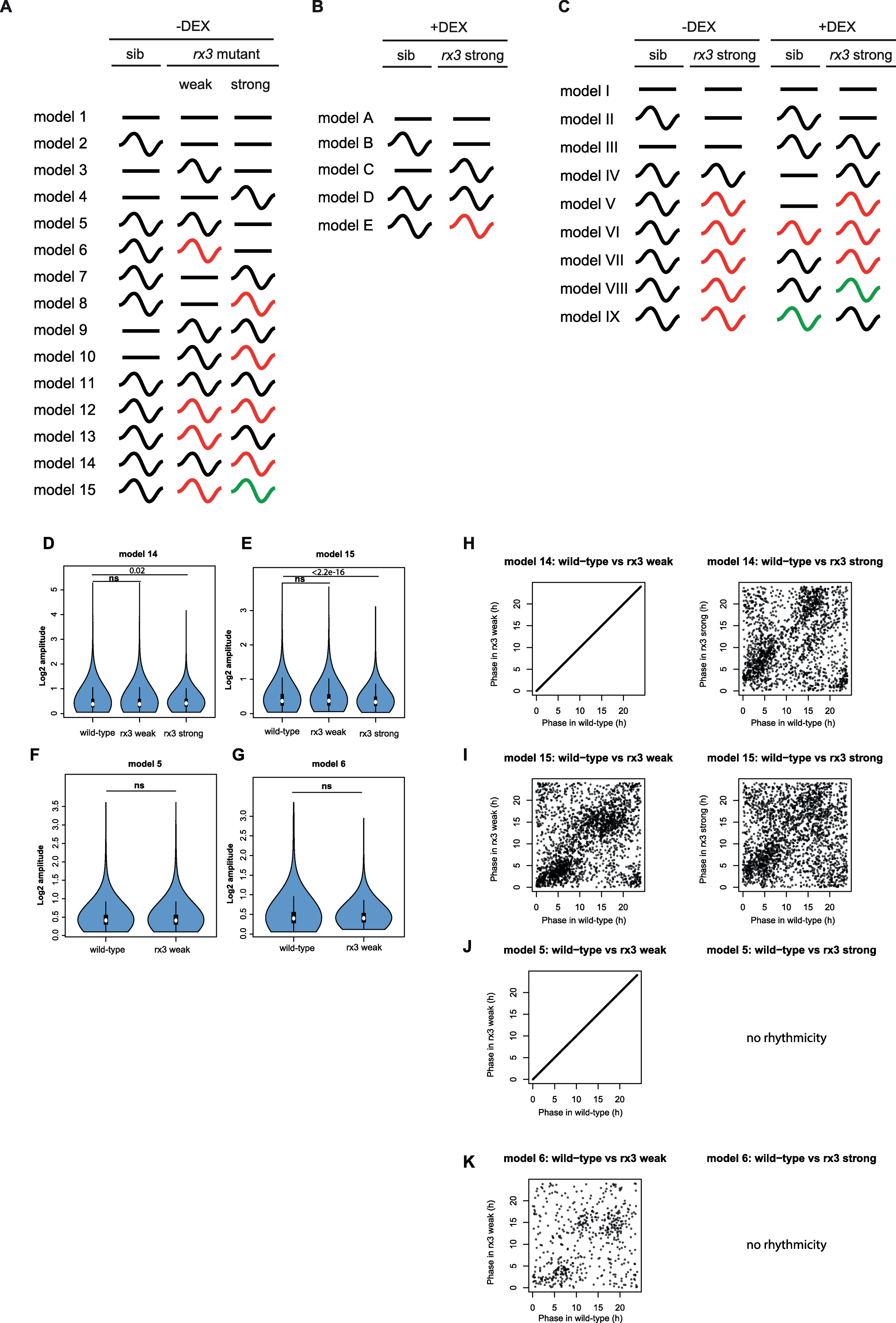

Supplement: S2 Fig — (A-C) Schematic overview of rhythmic patterns of all models. Black curved line: oscillation with same phase and amplitude as wild-type, black straight line: no oscillation, red or green curved lines: oscillation with a phase or amplitude different from wild-type. The green curved line indicates an oscillation with an amplitude and/or phase that is both different from wild-type (black) and from the other changed oscillation (red). (A) Untreated transcriptome models, (B) DEX treated transcriptome models, (C) metabolite models. (D-E). Violin plots of amplitudes across all genes belonging to model 14 (D), model 15 (E), model 5 (F) and model 6 (G), comparing all phenotypes still showing rhythmicity. (H-K) Phase distribution of wild-type and rx3 weak (left) or wild-type and rx3 strong (right) for model 14 (H), model 15 (I), model 5 (J) and model 6 (K). “no rhythmicity” = no comparison can be made, as the rx3 strong phenotype is arrhythmic. (TIF) [file pgen.1006512.s002.tif]

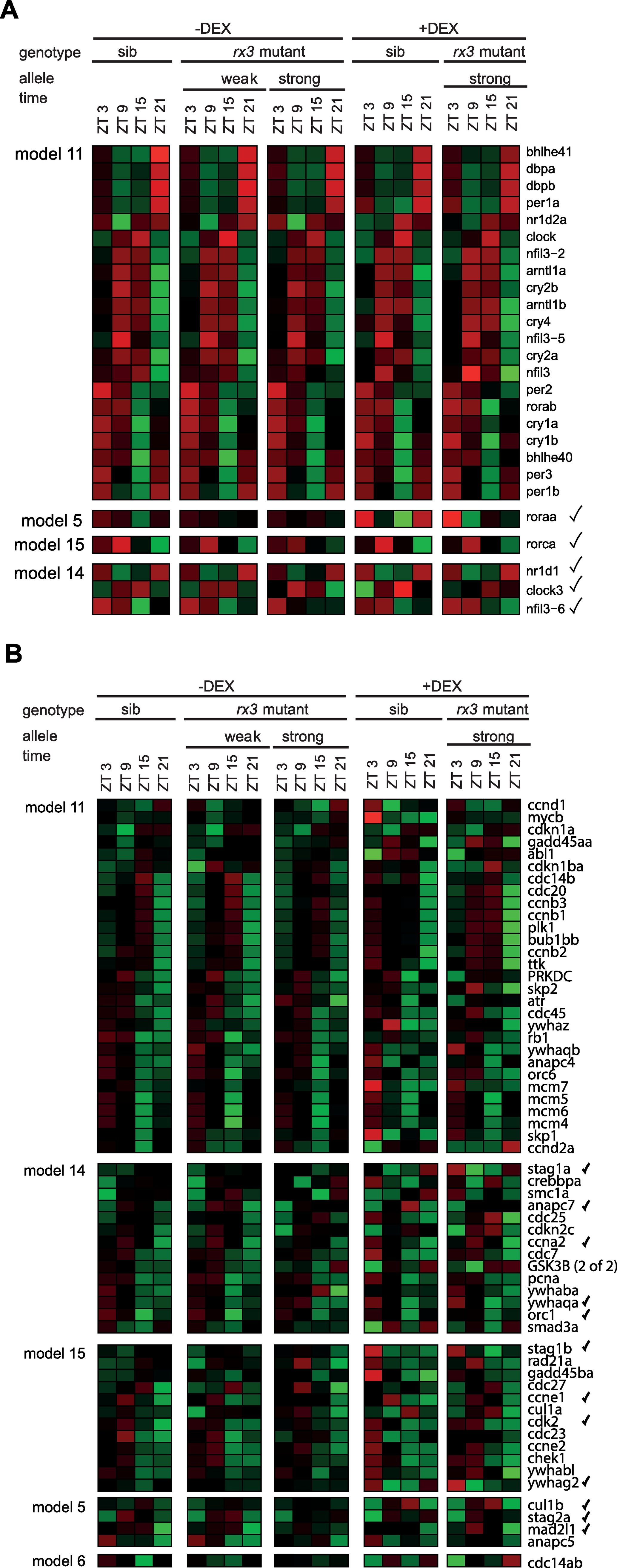

Supplement: S3 Fig — Heatmaps depicting mRNA levels of circadian clock genes (A) and cell cycle genes (B) in samples of the indicated conditions and time points. Genes are sorted according to their classifications within models (11, 5, 6, 14, 15). Expression values are colour-coded with green and red for low and high relative expression levels, respectively. Expression levels are normalized by the mean of each gene within each treatment/phenotype combination. Check marks indicate genes whose deregulated expression in rx3 strong mutants is rescued by DEX treatment. (TIF) [file pgen.1006512.s003.tif]

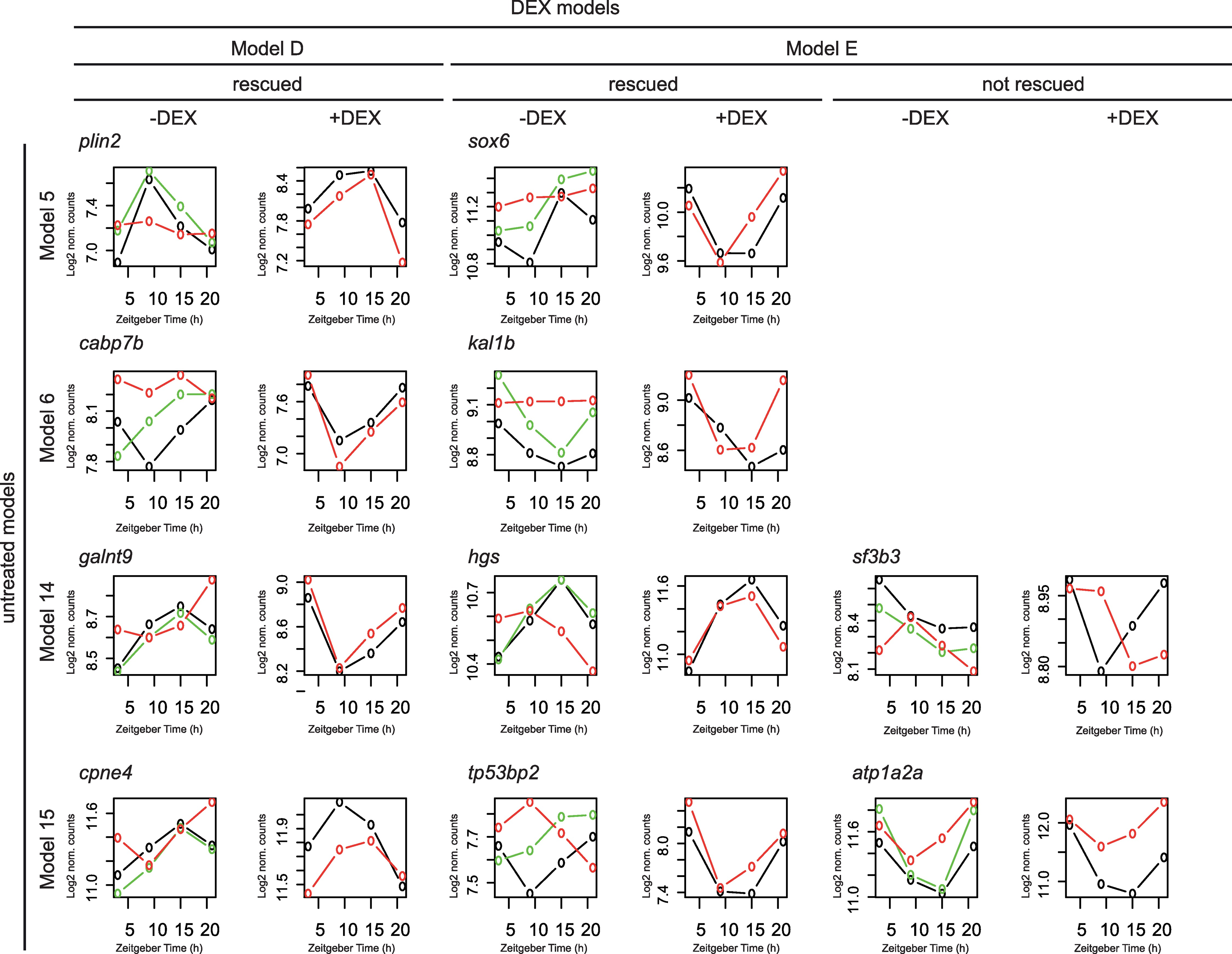

Supplement: S4 Fig — Red curves: rx3 strong, green curves: rx3 weak, black curves wild-type siblings. (TIF) [file pgen.1006512.s004.tif]

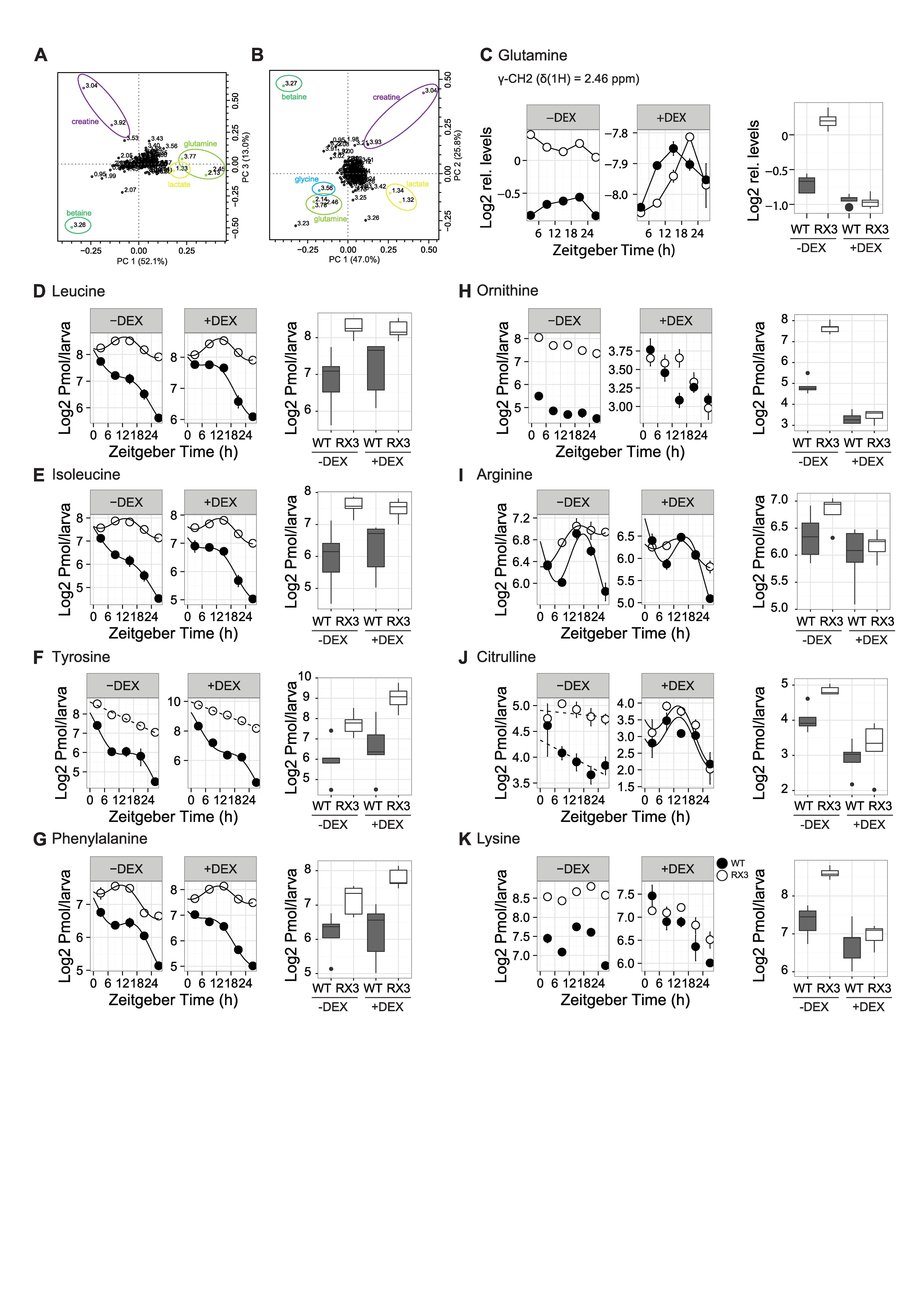

Supplement: S5 Fig — (A,B) Principal component analysis (PCA) loading plots of the NMR spectra of untreated (A) and DEX treated (B) samples. Color-coding indicates metabolites which correspond to the spectral peaks contributing the most to the first three principal components (PC1-3, % indicates contribution to total variance): creatine (purple), glutamine (light green), lactate (yellow), betaine (dark green), glycine (blue). (C) Left: Temporal profile of glutamine levels as determined by NMR analysis in wild-type (black) and rx3 strong mutants (white) in the absence or presence of dexamethasone (DEX). Error bars indicate SEM. Right: Tukey boxplot depicting glutamine levels at all examined time points in wild-type siblings (WT, white) and rx3 strong mutants (RX3, grey) in the absence or presence of DEX. (D-K) Temporal profiles of the indicated branched chain amino acid (BCAA; D and E), aromatic amino acid (AAA; F and G), ornithine-urea cycle (OUC) amino acid (H, I, J) and lysine levels (K) determined by UPLC-FLR and IC-CD analysis in wild-type (black) and rx3 strong mutants (white) in the absence or presence of dexamethasone (DEX). Lines fitted by linear regression shown as continuous if statistical models indicate rhythmicity and as broken if the fit was non-rhythmic. When the model assignment was “ambiguous”, no fit is indicated. Error bars indicate SEM. Right: Tukey boxplots depicting metabolite levels at all examined time points in wild-type siblings (WT, black) and rx3 strong mutants (RX3, white) in the absence or presence of DEX. (TIF) [file pgen.1006512.s005.tif]

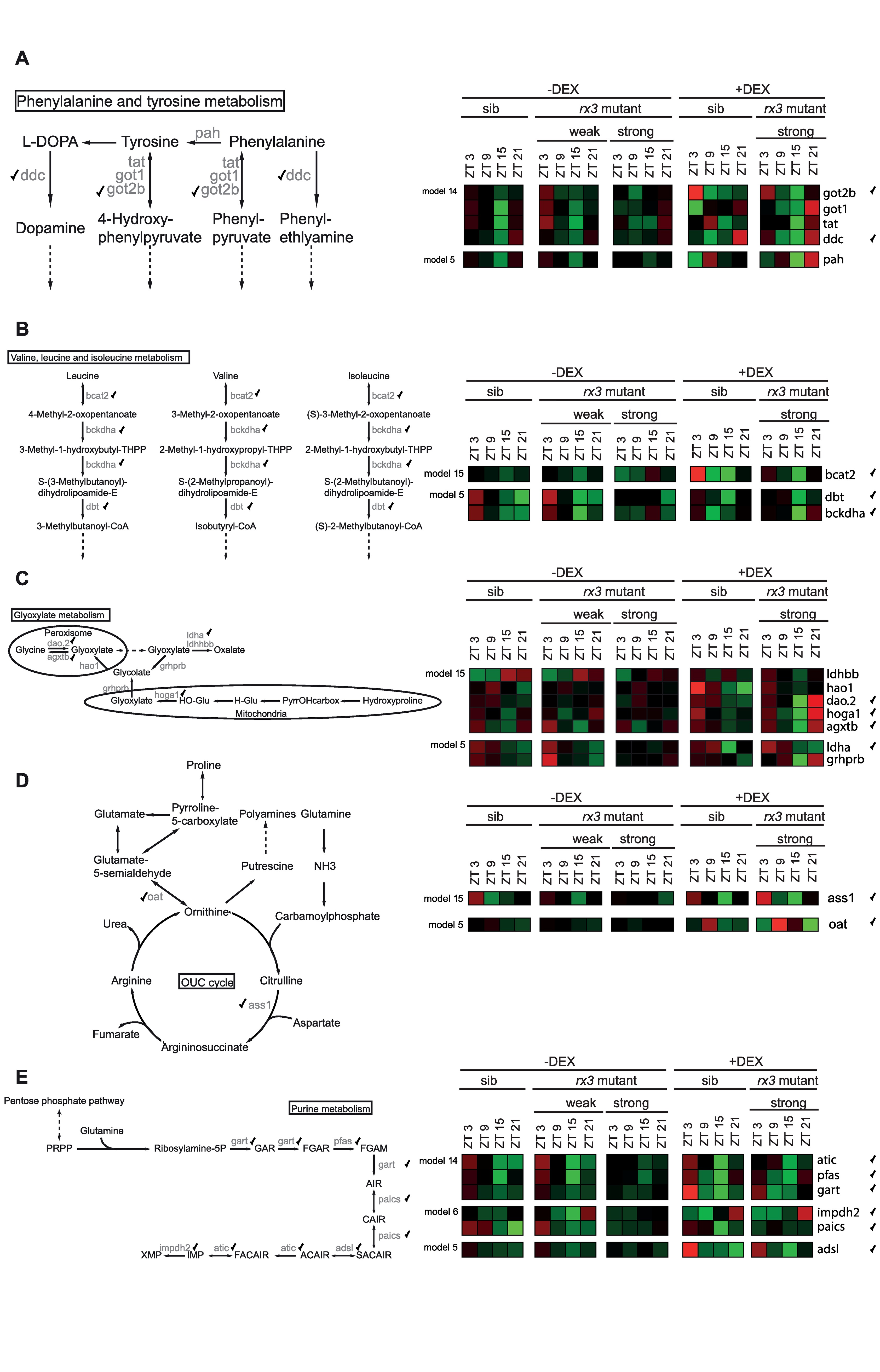

Supplement: S6 Fig — Left panels: Schematics of metabolism pathways indicating steps where genes with deregulated mRNA levels in rx3 strong mutants are involved. Right panels: Heatmaps of normalized gene expression levels of deregulated genes arranged according to model classification. Expression values are color-coded with green and red for low and high relative expression levels, respectively. Check marks indicate genes whose deregulated expression in rx3 strong mutants is rescued by DEX treatment. (A) Phenylalanine and tyrosine metabolism. (B) Valine, leucine, and isoleucine metabolism. (C) Glyoxylate metabolism. HO-Glu, 4-hydroxy-2-oxoglutarate; H-Glu, 4-hydroxy-glutamate, PyrrOHcarbox, pyrroline-5-carboxylate. Scheme following Salido et al. [17]. (D) Ornithine urea cycle (OUC). (E) Purine metabolism. (TIF) [file pgen.1006512.s006.tif]

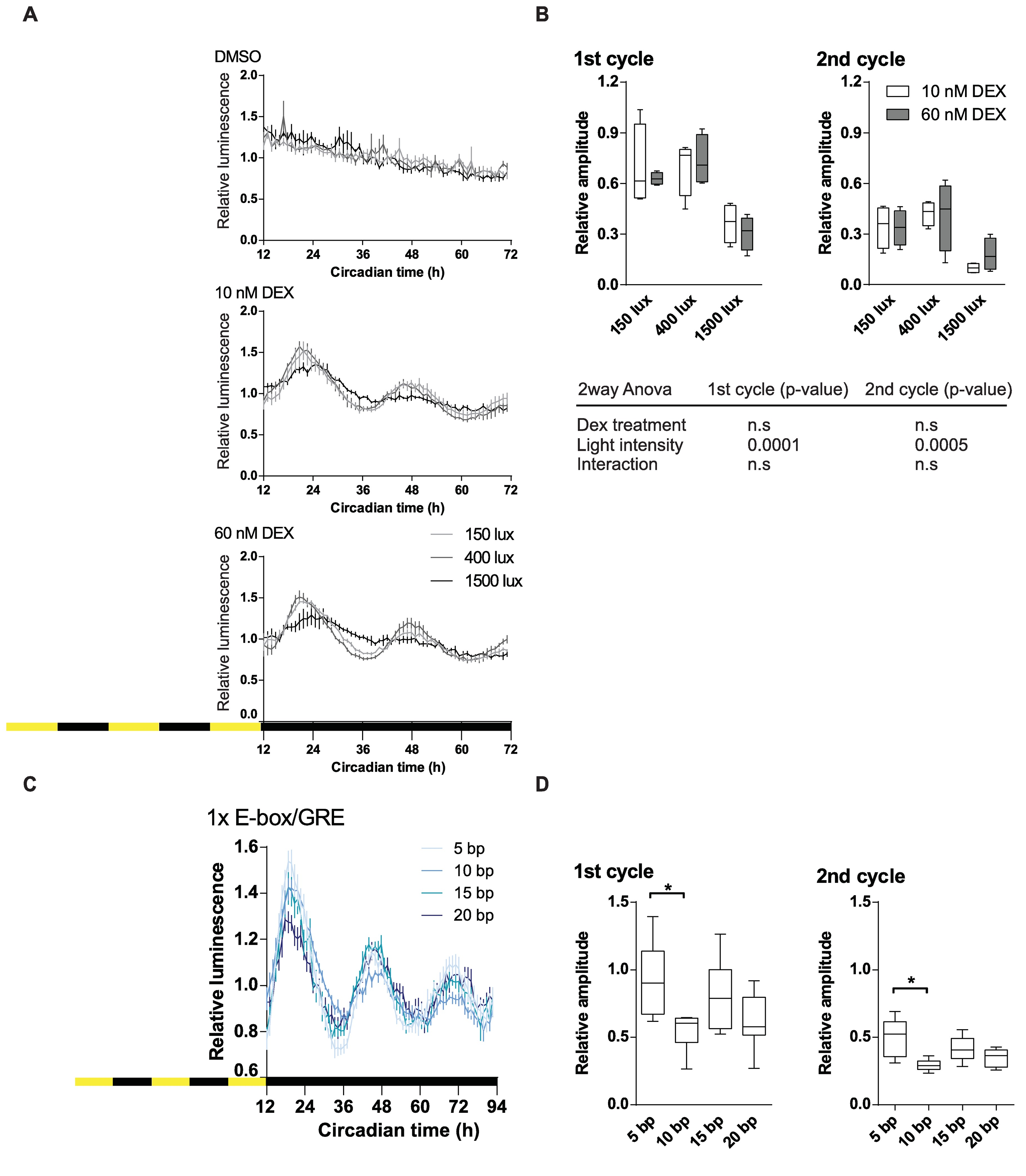

Supplement: S7 Fig — (A,B) Light intensity effects. (A) Bioluminescence traces recorded from zebrafish cells expressing the 1x E-box/GRE reporter constructs under different concentrations of DEX (10 nM, 60 nM) or DMSO as a control. Cells were entrained with 12 h light (yellow) / 12 h dark (black) cycles using different light intensities (150 lux, 400 lux, 1500 lux), then bioluminescence was recorded under constant darkness. (B) Tukey boxplots depicting the relative amplitudes of the first and the second cycle of the monitored bioluminescence oscillations shown in A. Relative amplitude is lower at the highest light intensity. Two-way ANOVA indicates significant effects on amplitude for light, but not for DEX treatment or an interaction between the two treatments. (C,D) Element spacing effects: (C) Bioluminescence traces recorded from zebrafish cells expressing the 1x E-box/GRE reporter constructs with different spacing (5 to 20 bp) between the E-box and GRE enhancer elements. (D) Tukey boxplots depicting the relative amplitudes of the first and the second cycle of the monitored bioluminescence oscillations shown in C. *, p < 0.05. n.s, not significant. (TIF) [file pgen.1006512.s007.tif]

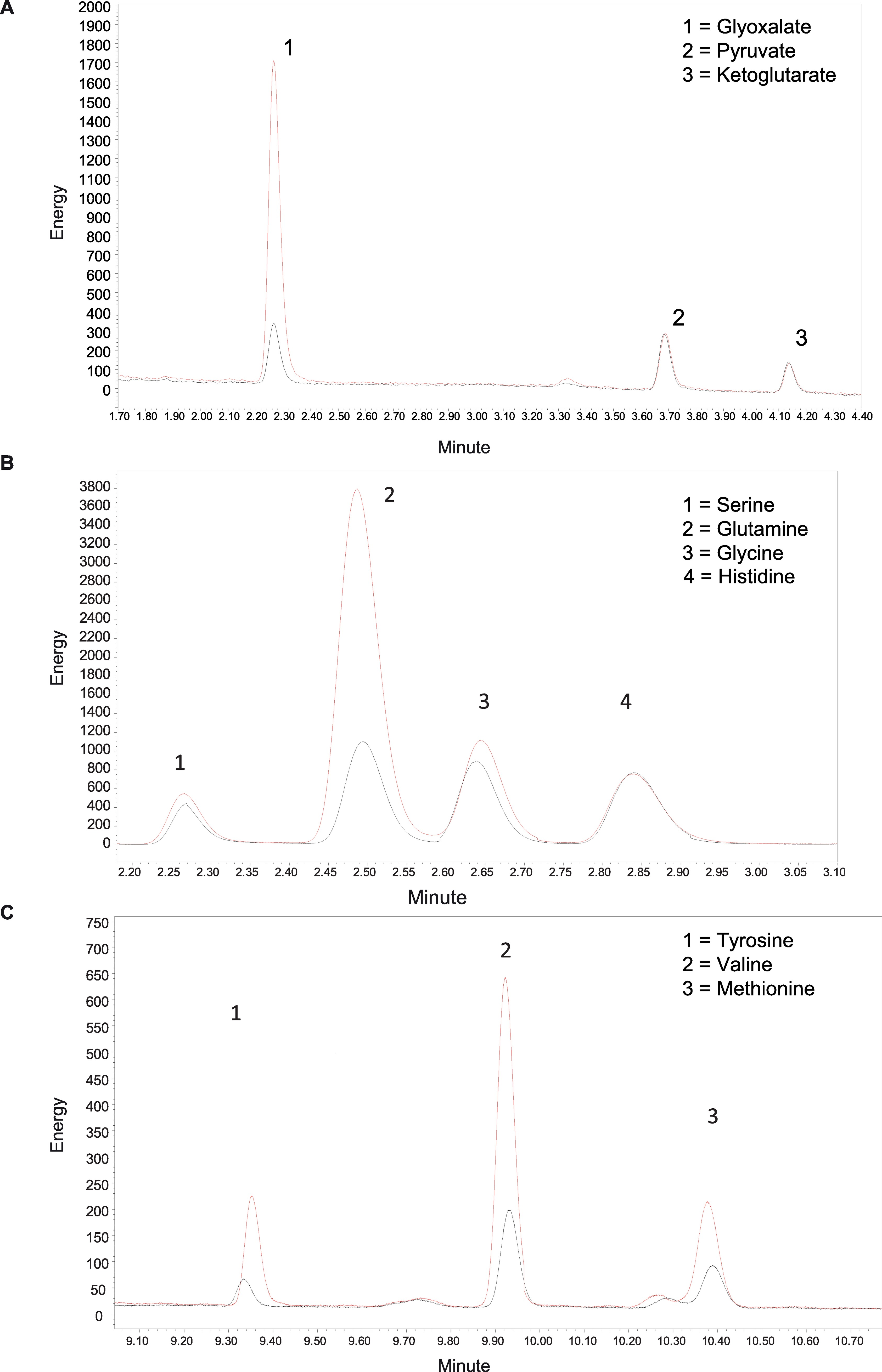

Supplement: S8 Fig — Shown are three examples for (A) glyoxylate, pyruvate, and ketoglutarate in samples at ZT9 (B) serine, glutamine, glycine, and histidine in samples at ZT3 (C) tyrosine, valine, and methionine in samples at ZT21. Black, wild-type; red, rx3 strong mutants (TIF) [file pgen.1006512.s008.tif]

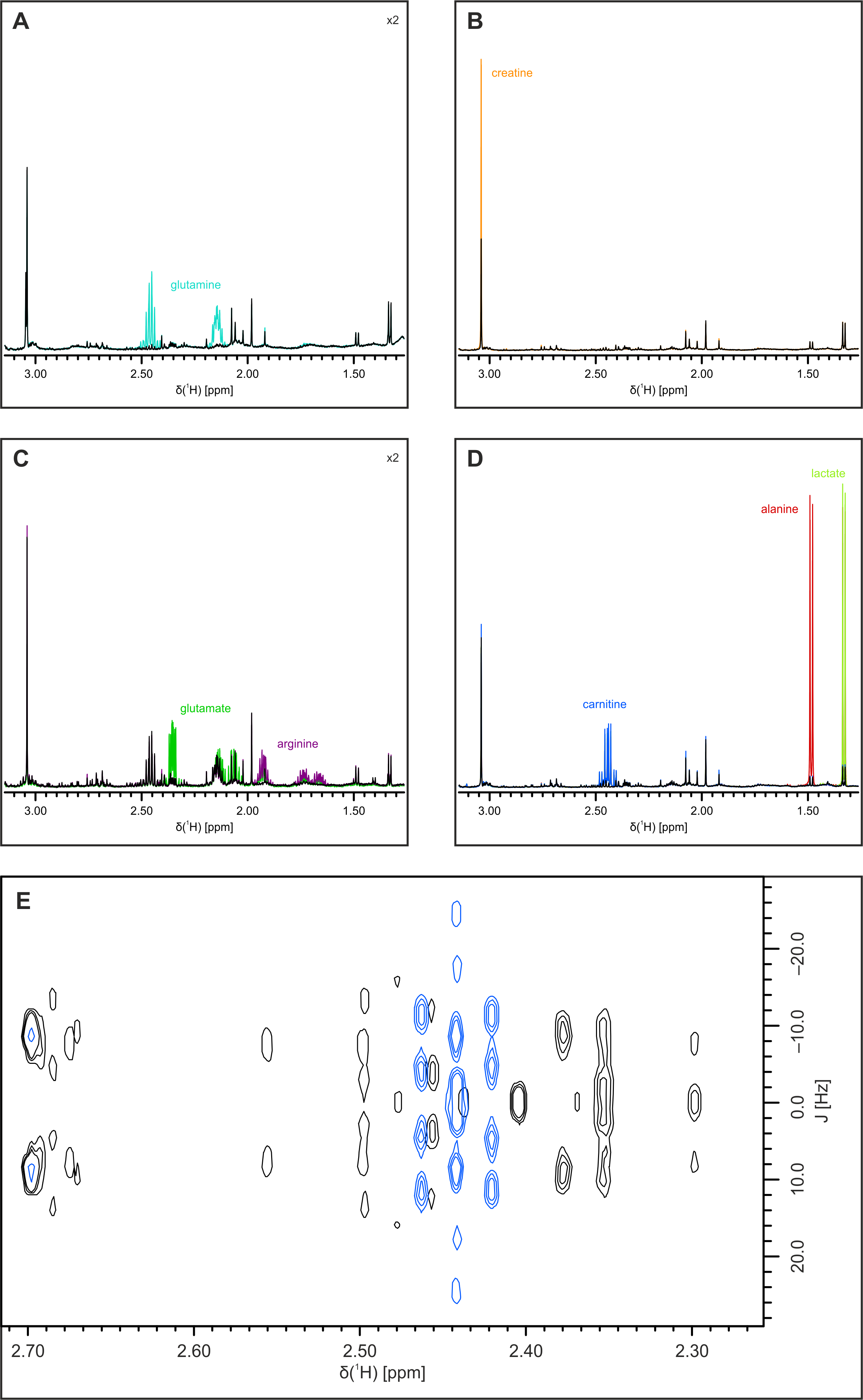

Supplement: S9 Fig — The shown spectra are extracts from wildtype siblings (D, E), rx3 strong mutants (C) and wildtype siblings treated with DEX (A, B) at ZT21 (A) or ZT3 (B-D). Extracts without spiked metabolites are always colored black. In some cases (C and D), samples were spiked two and three times with known compounds, as clear identification of the corresponding signals was still possible. Exemplary spectra of zebrafish extract with added glutamine (turquoise), creatine (orange), arginine (purple), glutamate (green), carnitine (blue), lactate (light green) and alanine (red) are shown. As the signals are frequently in crowded areas, spiking is necessary because comparison with databases would be inconclusive. The spiked carnitine signals in this region do not overlap with signals in the pure extract. For clarification, the excerpt of the corresponding J-resolved spectrum with easily identifiable multiplet patterns for carnitine is shown as well (E). (PNG) [file pgen.1006512.s009.png]
